# Simple prerequisite of presequence for mitochondrial protein import in the unicellular red alga *Cyanidioschyzon merolae*

**DOI:** 10.1242/jcs.262042

**Published:** 2024-07-23

**Authors:** Riko Hirata, Yuko Mogi, Kohei Takahashi, Hisayoshi Nozaki, Tetsuya Higashiyama, Yamato Yoshida

**Affiliations:** ^1^ Department of Biological Sciences, Graduate School of Science, The University of Tokyo, Tokyo 113-0033, Japan; ^2^ Biodiversity Division, National Institute for Environmental Studies, Ibaraki 305-8506, Japan; ^3^ Japan Science and Technology Agency (JST), PRESTO, Tokyo 113-0033, Japan

**Keywords:** Mitochondrial presequence, Mitochondrial protein import, Monomitochondrial eukaryote, Unicellular red alga

## Abstract

Mitochondrial biogenesis relies on hundreds of proteins that are derived from genes encoded in the nucleus. According to the characteristic properties of N-terminal targeting peptides (TPs) and multi-step authentication by the protein translocase called the TOM complex, nascent polypeptides satisfying the requirements are imported into mitochondria. However, it is unknown whether eukaryotic cells with a single mitochondrion per cell have a similar complexity of presequence requirements for mitochondrial protein import compared to other eukaryotes with multiple mitochondria. Based on putative mitochondrial TP sequences in the unicellular red alga *Cyanidioschyzon merolae*, we designed synthetic TPs and showed that functional TPs must have at least one basic residue and a specific amino acid composition, although their physicochemical properties are not strictly determined. Combined with the simple composition of the TOM complex in *C. merolae*, our results suggest that a regional positive charge in TPs is verified solely by TOM22 for mitochondrial protein import in *C. merolae*. The simple authentication mechanism indicates that the monomitochondrial *C. merolae* does not need to increase the cryptographic complexity of the lock-and-key mechanism for mitochondrial protein import.

## Teaser

**Summary:** An N-terminal peptide with a specific amino acid composition and very few basic residues is sufficient for mitochondrial protein targeting in *Cyanidioschyzon merolae*.

## INTRODUCTION

Evolved from a free-living α-proteobacterial ancestor via an endosymbiotic event, the mitochondrion has its own genome and gene expression system (Gray et al., 1999). However, most genes encoding mitochondrial proteins are now encoded in nuclear genomic DNA and 99% of mitochondrial proteins are synthesized by cytosolic ribosomes (Chacinska et al., 2009; Sickmann et al., 2003). Due to the compartmentalization by membranes, nascent mitochondrial precursor proteins are secreted into mitochondria according to the embedded information in their peptide sequence (Pfanner et al., 2019; Schmidt et al., 2010). Except for mitochondrial membrane proteins, the signature for transportation into mitochondria can be found in the N-terminal peptide sequence of precursor proteins called the presequence or targeting peptide (TP) (Endo and Yamano, 2009). The precursor proteins that have the TP are carried into the mitochondrion through the mitochondrial protein translocator of the outer membrane (called the TOM complex) (Araiso et al., 2019; Shiota et al., 2011, 2015; Su et al., 2022). Then, precursor proteins are imported into the matrix by the function of the TIM23 complex (Sim et al., 2023; Yamamoto et al., 2002; Zhou et al., 2023). The mitochondrial protein import system is widely conserved through eukaryotes, suggesting that it evolved in their last common ancestor.

To distinguish the nascent proteins that should be secreted into the mitochondrion from others, the mitochondrial targeting sequence has several characteristic features. Mitochondrial TPs usually contain 20 to 60 amino acids and tend to fold into amphiphilic α-helices (Calvo et al., 2017; Carrie et al., 2015; Gavel et al., 1988; Huang et al., 2009). Their amino acid composition is generally enriched in alanines, leucines, lysines and arginines. In particular, it is considered that the presence of arginine residues and the resultant positive charge in the presequence determines mitochondrial targeting (Vögtle et al., 2009; von Heijne et al., 1989). The reason for the overall enrichment of basic amino acids is thought to be that the positive charge of the TP facilitates passage through the electrochemical gradient across the inner mitochondrial membrane generated by the mitochondrial electron transport chain (Garg and Gould, 2016).

Despite these features being well known in the mitochondrial targeting sequences throughout eukaryotes, the common motif has not been identified and amino acid sequences differ even in the same organism. Although the targeting sequence appears to be a cryptic sequence, significant biochemical features of the presequence indicate that the organelle destinations of each protein are controlled and follow uncharacterized rules in the eukaryotic cell. Given that each organism shows different trends in the mean length and amino acid composition of the presequences, there are countless functional sequences as the presequence and the complexity of the presequences might correlate with the complexity of cell structure such as multicellularity.

**Fig. 1.**
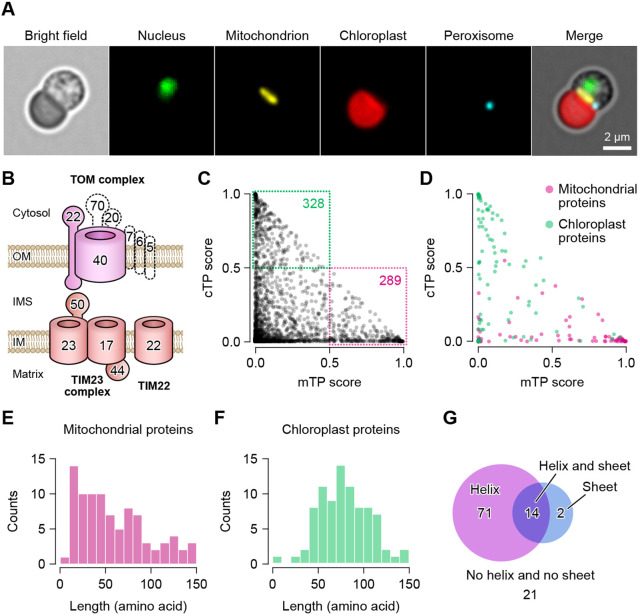
**Genomic information of mitochondrial and chloroplast proteins in *Cyanidioschyzon merolae*.** (A) Fluorescence images of a *C. merolae* cell. The *ACTIN* knockout cell was used for the imaging (Tanaka et al., 2021). The nucleus, mitochondrion, chloroplast and peroxisome were visualized by Cas9–Venus, mScarlet, chlorophyll autofluorescence and mCerulean3, respectively. Images are representative of more than three independent experiments. (B) The mitochondrial translocater of the outer mitochondrial membrane (TOM) complex and the inner mitochondrial membrane (TIM) complex. The TOM complex is composed of the β-barrel protein TOM40, α-helical membrane-integrated receptors TOM20, TOM22 and TOM70, and the regulators TOM5, TOM6 and TOM7. Proteins which are not identified in the *C. merolae* protein-coding genes are illustrated with dashed lines. OM, outer membrane; IMS, intermembrane space; IM, inner membrane. (C,D) Scatterplot comparisons of mitochondrial targeting peptide (mTP) and chloroplast targeting peptide (cTP) scores for all ORFs (4803 proteins) (C) and for well-characterized mitochondrial (113 proteins) and chloroplast proteins (97 proteins) (D). Prediction scores for all ORFs are shown in Table S1 and the list of mitochondrial and chloroplast proteins is given in Tables S2 and S3. (E,F) Histograms of the length of presequences for the mitochondrion (E) and the chloroplast (F). (G) Venn diagram showing the classification of mitochondrial presequences containing α-helices (magenta), β-sheets (blue), both α-helices and β-sheets (purple), and no α-helices or β-sheets. See also Figs S1 and S2.

In this study, to understand the prerequisite of the TP for mitochondrial targeting, we experimentally assessed protein targeting function of various types of synthetic TPs (synTPs) using the unicellular alga *Cyanidioschyzon merolae*. The *C. merolae* cell contains only one mitochondrion, which is easily identified by its shape and intracellular localization by fluorescence microscopy (Kuroiwa, 1998; Matsuzaki et al., 2004; Nozaki et al., 2007) (Fig. 1A). In addition, established gene-targeting techniques using fluorescent reporters can be used to test whether the engineered TP has the ability to target a fluorescent protein (FP) or FP-fused protein to the mitochondrion of *C. merolae* (Fujiwara et al., 2015; Imamura et al., 2009; Ohnuma et al., 2008; Tanaka et al., 2021). Through a series of *in vivo* and *in silico* experiments, we showed that an N-terminal peptide with a specific amino acid composition and very few basic residues fulfils the requirement for mitochondrial protein targeting. Thus, a key with a simple structure can open the mitochondrial protein gate in the monomitochondrial *C. merolae*.

## RESULTS

### Genome analysis of the presequences in *C. merolae*

The fact that only 4803 genes are encoded in the nuclear genome and 99.9% of the protein-coding genes are single-exon genes suggests a simple proteome in *C. merolae* (Matsuzaki et al., 2004; Nozaki et al., 2007). Furthermore, as only two components, TOM40 and TOM22, in the TOM complex are characterized in the genome, the mitochondrial protein targeting system in *C. merolae* is likely to be functionally limited than that in other eukaryotic cells (Fig. 1B). To reveal the minimal requisite as the functional TP for mitochondrial protein targeting, we first computationally evaluated all amino acid sequences encoded in the nuclear genome by using a deep learning model-based presequence prediction tool, TargetP2.0 (Armenteros et al., 2019). The protein-encoding open reading frames (ORFs) were classified based on their mitochondrial TP (mTP) and chloroplast TP (cTP) scores: 336 putative mitochondrial proteins (289 proteins with an mTP score >0.5) and 349 putative chloroplast proteins (328 proteins with a cTP score >0.5) were identified (Fig. 1C; Table S1). To assess the predictability of the results, we confirmed the prediction scores for mitochondrial and chloroplast proteins that are well characterized in other organisms or experimentally confirmed to localize to the mitochondrion or the chloroplast in *C. merolae* (Mori et al., 2016; Moriyama et al., 2014a,b) (Tables S2 and S3). The recalls of protein targeting sequences for mitochondrial proteins (113 proteins) and chloroplast proteins (97 proteins) were 43.4% (49 proteins with a mTP score >0.5) and 41.2% (40 proteins with a cTP score >0.5), respectively (Fig. 1D). Given that the recall of presequence prediction for mitochondrial and chloroplast proteins in other organisms by TargetP2.0 was approximately 80–86% (Armenteros et al., 2019; Imai and Nakai, 2020), the lower prediction scores for *C. merolae* protein targeting sequences suggest that the mitochondrial targeting system in *C. merolae* not only shares basic similarities with those in other eukaryotes, but also has some differences.

To understand the characteristics and principles of mitochondrial TPs in *C. merolae*, we next compared the length of the putative mitochondrial and chloroplast TPs. In the comparison, TP regions in mitochondrial and chloroplast proteins were presumed by protein sequence alignment analysis (see Materials and Methods) and we omitted proteins for which the length of the putative TPs was shorter than ten or longer than 150 amino acids from the analysis. The lengths of mitochondrial and chloroplast TPs were 59.3±23.4 and 78.4±17.3 (mean±s.d.), respectively (Fig. 1E,F). Additionally, our dataset for mitochondrial TPs showed that 75.2% of mitochondrial TPs (85/113 proteins) are α-helical polypeptides (Fig. 1G; Figs S1 and S2). Thus, similar to the results in other organisms, the mitochondrial TP is typically shorter than the chloroplast TP and the α-helical structure is the significant feature of the mitochondrial TP even in the *C. merolae* cell with the simplest organelle composition.

### *In vivo* analysis of mitochondrial targeting property by fluorescence reporter assay

**Fig. 2.**
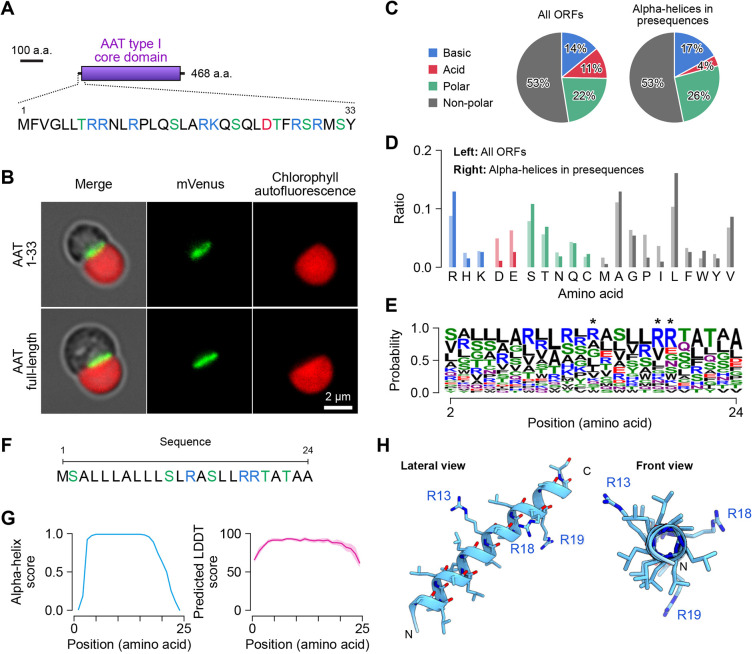
**Synthetic mitochondrial presequence.** (A) Scheme of the 1–33 amino acid sequence of aspartate aminotransferase (AAT, CMC148C). (B) Fluorescence images of AAT 1–33 and AAT full-length fused with mVenus. Fluorescence signals for mVenus are shown in green and chlorophyll autofluorescence is shown in red. See also Fig. S3. Images are representative of three independent experiments. (C) Comparisons of amino acid compositions in all ORFs and α-helices in mitochondrial presequences. The α-helices in mitochondrial presequences are identified by structural simulation using the AlphaFold program. See also Table S4 for sequences. (D) Distribution of amino acids in all ORFs (individual bars on the left) and α-helices in presequences (individual bars on the right). (E) An amino acid sequence logo of α-helices in presequences. Asterisks indicate the arginine residues that were adopted in the synthetic presequence. (F) Sequence of the synthetic presequence of 24 amino acids. (G) Structural prediction score for α-helix (left) and local distance difference test (LDDT) score (right) of the synthetic presequence. (H) A simulated structure of the synthetic presequence in lateral and front views.

To verify whether the single α-helical polypeptide could work as a TP in *C. merolae*, an α-helix region (1–33 amino acids) or the full length (1–468 amino acids) of aspartate aminotransferase (AAT, CMC148C) was fused with the yellow fluorescent protein mVenus (Nagai et al., 2002) and introduced into the cells as a representative example (Fig. 2A,B; Fig. S3). The resultant transformants expressing either in AAT 1–33–mVenus or AAT full-length–mVenus emitted fluorescence signals of the mVenus reporter in the mitochondrion. The result suggests that the α-helical polypeptide is one of the minimal requisites as a functional presequence for mitochondrial protein targeting in *C. merolae*.

Next, we analyzed the amino acid compositions of the α-helices of mitochondrial TPs. For the analysis, 31 single α-helical polypeptides, which are less than 25 amino acids long, in TPs were evaluated (Table S4). As a result, we identified that the α-helical polypeptides were composed of 17.3% basic residues, 3.6% acidic residues, 26.2% polar uncharged residues and 52.9% of nonpolar residues (Fig. 2C, right). Given that the average amino acid composition of all *C. merolae* ORFs is 11.2% basic residues, 14.0% acidic residues, 22.2% polar uncharged residues and 52.5% nonpolar residues (Fig. 2C, left), although the decrease in the proportion of acidic residues was remarkable, the overall trend was not extremely skewed in the mitochondrial TPs. More clear differences were found not in the chemical characteristics, but in the amino acid compositions. Although the aspartic acid, glutamic acid, proline and isoleucine residues are scarce, the arginine, serine, threonine, alanine, leucine and valine residues are abundant in the mitochondrial TPs (Fig. 2D). Of these abundant residues, it is known that alanine, arginine and leucine have high α-helical propensities (Pace and Scholtz, 1998). As the mean hydrophobic moment in the α-helical polypeptides was 0.288±0.13 μH (±s.d.) (Table S4), the TP has very weak amphiphilicity. Also, owing to the low composition of acidic residues, the average charge of the α-helices was estimated to be 2.80±1.76 at pH 7.0 (Table S4). Thus, the mitochondrial TP has a specialized amino acid composition compared with that of other polypeptides and a tendency to form α-helical structure with weak amphiphilicity and weak positive charge in *C. merolae*.

### Mitochondrial targeting property of a designed presequence

**Fig. 3.**
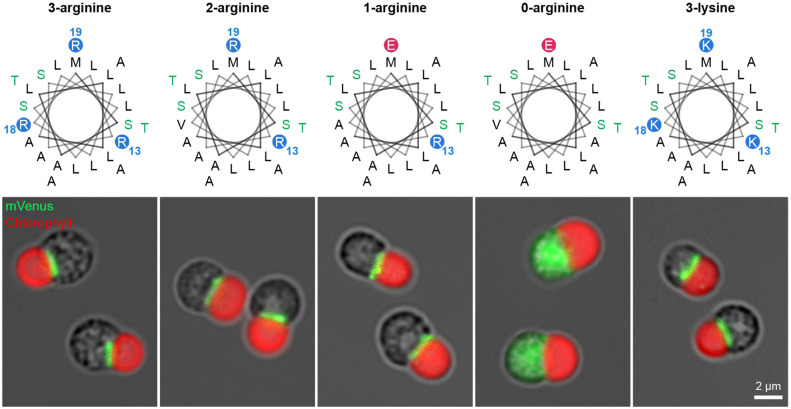
**Effects of the number of basic residues in the synthetic presequence.** The helical wheel diagrams for amino acid sequences of the synthetic presequences containing three, two, one or zero arginine residues and three lysine residues. Representative images of cells from three independent experiments are shown below each helical wheel.

To further investigate the basal prerequisites of the mitochondrial TP, we designed a synTP by linking the most frequent amino acid residue at each position in the α-helices (Fig. 2E). In order to make the charge similar to that of the endogenous TPs, the two arginine residues were replaced with leucine and serine residues, respectively. As a result, the synTP contains three arginine residues in the 24 amino acids (Fig. 2F). Computational simulations of both the secondary and tertiary structure indicated that the synTP forms a single α-helical structure (Fig. 2G,H). By introducing synTP-fused mVenus into the cell, we detected the fluorescence signal in the mitochondrion and concluded that the designed TP has the mitochondrial targeting property (Fig. 3, left). As it is well known that multiple basic residues in the TP are required for protein targeting into the mitochondrion (Gavel et al., 1988), we investigated the requirement of the minimum number of basic residues for mitochondrial protein targeting in *C. merolae*. To investigate this, arginine residues in the synTP were progressively replaced by other residues. Interestingly, although computational simulations predicted that modified synTPs containing less than two arginine residues would lose the ability to translocate to the mitochondrion (Table S5), transformants expressing modified synTP fused to mVenus showed that even a single arginine residue in the TP fulfils the role for mitochondrial targeting (Fig. 3). In addition, we confirmed that lysine residues are exchangeable for arginine residues in the TP (Fig. 3, right).

### Identification of physicochemical properties for a functional presequence

**Fig. 4.**
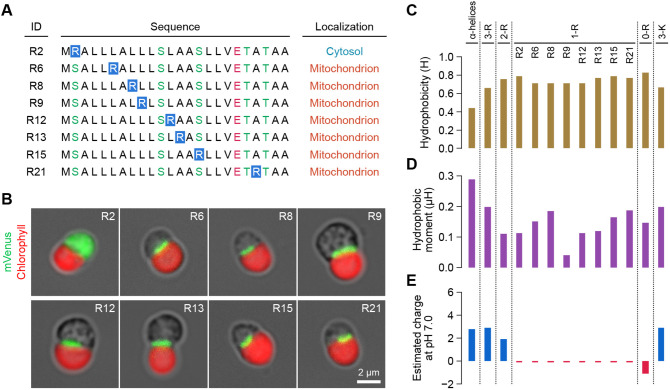
**Arginine scanning analysis and physicochemical properties of the modified synthetic presequences.** (A) Arginine scanning of the modified synthetic presequence. (B) Fluorescence images of each modified synTP^1R^–mVenus. Images are representative of three independent experiments. (C–E) Physicochemical properties of each synthetic presequence. See also Table S5.

Generally, mitochondrial TPs contain an arginine residue near the protease cleavage site with a sequence motif of R-X-/-X or R-X-F/T/L-/-A/S-X (Heidorn-Czarna et al., 2022), indicating that not only the net charge but also position of arginine residues would affect the mitochondrial targeting property. Based on this assumption, we investigated the effect of the localization of the single arginine residue in the synTP^1R^ by an arginine-scanning approach. The position of a single arginine residue at the +2, +6, +8, +9, +12, +13, +15 or +21 position was examined (Fig. 4A). After the series of analyses, we found that the position of the single arginine residue in synTP^1R^ is broadly permissible in the α-helix, but the synTP^1R^ containing an arginine residue at the +2 position relative to the initial methionine residue lost the mitochondrial targeting property and the mVenus reporter localized in the cytosol similar to synTP^0R^ (Fig. 4B). Taken together, an arginine residue in the α-helix can impart function to the mitochondrial TP and the position of the arginine residue is allowed in a broad region of the helical structure except for the flanking position.

By changing either the number of arginine residues or the position of the arginine residue, the physicochemical properties of synTP are drastically altered (Fig. 4C–E). As the hydrophobicity and hydrophobic moment are acceptable in a broad range (0.388 to 0.747 H and 0.246 to 0.101 μH) as functional TPs, the importance of these factors for protein targeting to the mitochondrion is not high. Furthermore, neither the hydrophobicity nor the hydrophobic moment of the mitochondrial TP in *C. merolae* has been characterized. More importantly, the *C. merolae* mitochondrial TP functioned normally even when the charge was negative. Although the results suggest that the net charge of the TP does not need to be positive for protein targeting to the mitochondrion, a modified TP without an arginine residue (synTP^0R^) lost the ability to target mitochondrial proteins (Fig. 3). It is therefore suggested that basic residues in the α-helix, with the exception of the flanking position, are essential for a functional TP, but their importance is not linked to the net charge of the TP.

### Verification of the mitochondrial targeting property of endogenous polypeptides sharing sequence similarity with the synTP

Our results showed that the specialized amino acid composition and the presence of a few basic residues in the α-helix are prerequisites for the functional TP in *C. merolae*; however, it is questionable whether these lax requirements are sufficient to distinguish mitochondrial protein precursors from other proteins *in vivo*. If the peptide sequence of the synTP is very specific in protein sequences and only endogenous mitochondrial TPs have sequence similarity with the synTP in *C. merolae*, the sequence would act as a key for the mitochondrial protein gate. In contrast, if the sequence has some similarity to other proteins, gene products with translation errors or genetic mutations that cause amino acid substitutions in each gene would easily lead to misdelivery of the protein to the mitochondrion. To address this issue, we evaluated the sequence similarity between the N-terminal peptides (1–24 amino acids) of all ORFs and the synTP using the BLOSUM30 matrix.

**Fig. 5.**
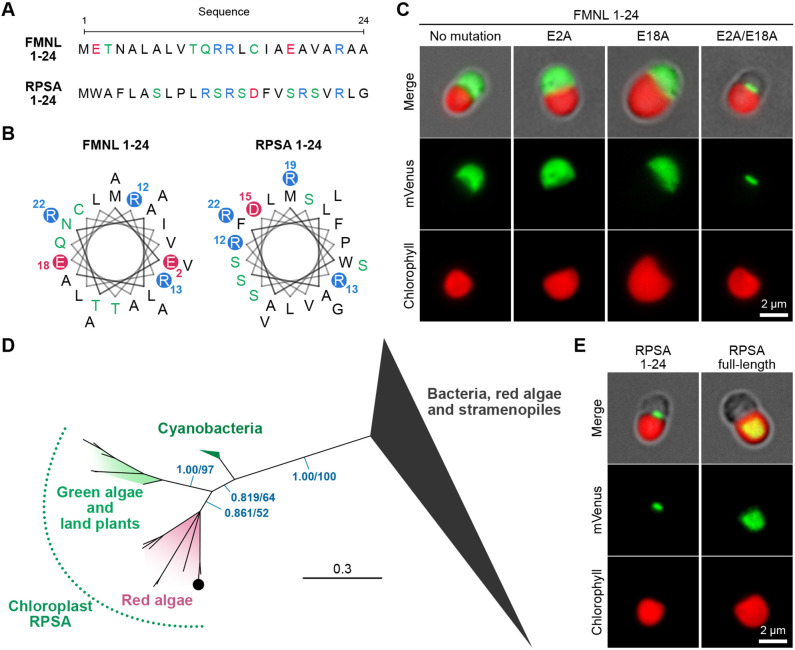
**Fluorescent reporter assay for putative presequences of FMNL and RPSA.** (A) Sequences of the first 24 amino acids of formin-like protein (FMNL) and r-protein S1 (RPSA). (B) Helical wheels for FMNL 1–24 and RPSA 1–24. (C) Fluorescence images of the N-terminal 1–24 peptide of FMNL fused with mVenus. Mutated FMNLs (E2A, E18A and E2A/E18A) fused with mVenus are also shown. (D) The Bayesian tree of chloroplast, mitochondrial and bacterial RPSA proteins (see Fig. S4 for the unabbreviated tree). Numbers on the left and right near branches indicate posterior probabilities of Bayesian inference and bootstrap values of the maximum likelihood method, respectively. Branch lengths are proportional to the evolutionary distances indicated by the scale bar. The *C. merolae* RPSA (CMM019C) is shown as the black circle. (E) Fluorescence images of RPSA 1–24 or RPSA full-length fused with mVenus. Images are representative of three independent experiments.

Consequently, not only mitochondrial proteins, but also many cytosolic and chloroplast proteins were highly scored (Table S6). For example, a hypothetical protein (CMD051C), a putative formin-like protein (FMNL, CMN049C) and a putative chloroplast ribosomal protein (r-protein) S1 (RPSA, CMM019C) were ranked first to third. The N-terminal peptides for FMNL and RPSA were also highly predicted to form an α-helical structure by local structure prediction, as was the case for synTP (Fig. 5A,B). FMNL, a cytosolic protein related to actin filament dynamics, and chloroplast RPSA, a 30S r-protein S1 in chloroplasts, are well studied, but it has not been reported that these proteins localize to mitochondria.

### Fluorescence reporter assay for the native or modified N-terminal polypeptides of FMNL

To determine whether these N-terminal peptides have mitochondrial targeting function, we performed the fluorescent reporter assay for the 1–24 amino acids of FMNL. As a result, the fluorescence signal for FMNL 1–24 fused to mVenus was identified in the cytosol, suggesting that FMNL 1–24 is similar to synTP but does not have a mitochondrial targeting property (Fig. 5C, left). As a notable difference between FMNL^1–24^ and synTP is the total number of acidic residues, we hypothesized that a negative charge derived from these residues prevents translocation of FMNL to the mitochondrion. Therefore, two acidic acid residues (E2 and E18) were progressively replaced by alanine residues. Using this approach, we found that a single substitution of glutamic acid residues did not affect the targeting property, but double mutation (E2A and E18A) conferred the targeting property to the mitochondrion (Fig. 5C). This demonstrates that a small number of amino acid substitutions can alter the destination of a protein *in vivo*.

### The evolutional origin and the targeting property of the N-terminus of RPSA

As a second example, we investigated the targeting property of the N-terminus of RPSA. Prior to the *in vivo* assay, we performed phylogenetic analysis to verify whether *C. merolae* RPSA (CMM019C) shares sequence similarity and is evolutionarily related to other chloroplast and cyanobacterial RPSAs. The results showed that cyanobacterial, green algal/land plant and red algal RPSAs containing the *C. merolae* RPSA formed a phylogenetic group and were separated from other bacterial and eukaryotic RPSAs (Fig. 5D; Fig. S4). The evolutionary relationship between *C. merolae* RPSA and cyanobacterial RPSA suggests that the gene product of CMM019C would function as a 30S r-protein S1 in the chloroplast, not in the mitochondrion. Next, we tested the targeting property of the N-terminal peptide sequence of the chloroplast RPSA using the fluorescent reporter assay. As a result, we found that the N-terminal peptide has the property to translocate to the mitochondrion but not to the chloroplast (Fig. 5E, left). We also confirmed that the full-length RPSA fused to mVenus translocated to the chloroplast (Fig. 5E, right). The results suggest that the N-terminus of RPSA has the same targeting property to the mitochondrion as that of the synTP, but an additional peptide sequence would convert it to a chloroplast TP.

## DISCUSSION

### The single-step authentication of mitochondrial preproteins in *C. merolae*

Through a series of *in vivo* and *in silico* experimental evaluations using synTPs, we showed the minimal prerequisite for protein targeting to the mitochondrion in *C. merolae*. The results indicated that functional TPs need to have some basic residues, at least one, in an α-helix comprising the specific amino acid composition, but the physicochemical properties of the net charge, hydrophobicity and hydrophobic moment do not seem to be strictly determined in *C. merolae*. The reason for the small number of basic residues in the TP could be explained by the characteristics of the TOM complex in *C. merolae*. Previous pioneering studies in fungi and animals indicated that the mitochondrial outer membrane proteins TOM20 and TOM22 identify mitochondrial TPs by multi-step authentication and introduce mitochondrial precursors into the mitochondrial intermembrane space (Abe et al., 2000; Araiso et al., 2019; Hanif Sayyed and Mahalakshmi, 2022; Nargang et al., 1998; Pfanner et al., 2019; Saitoh et al., 2007; Shiota et al., 2011). During the process, precursor proteins in the cytosol are initially detected and captured by TOM20 on the mitochondrial outer membrane via the hydrophobic interaction between the hydrophobic groove on the TOM20 surface and the hydrophobic site of the amphipathic helix of the TP (Abe et al., 2000; Saitoh et al., 2007). Then, TOM22 interacts with the TP via the electrostatic interaction between the negatively charged site of TOM22 and the positively charged site of the helix in the TP (Nargang et al., 1998; Shiota et al., 2011; Su et al., 2022). After the verification by TOM20 and TOM22, precursors are delivered to the β-barrel protein transporter TOM40 and the TIM complex. Although a TOM22 homologue has been identified in *C. merolae*, there is no sequence encoding TOM20 in the genome (Fig. 1B). Given that hydrophobicity is not strictly required for the functional TP (Fig. 4C), combined with the absence of TOM20 in the genome, verification of the mitochondrial precursors in *C. merolae* would be performed by TOM22 alone as a single-step authentication.

**Fig. 6.**
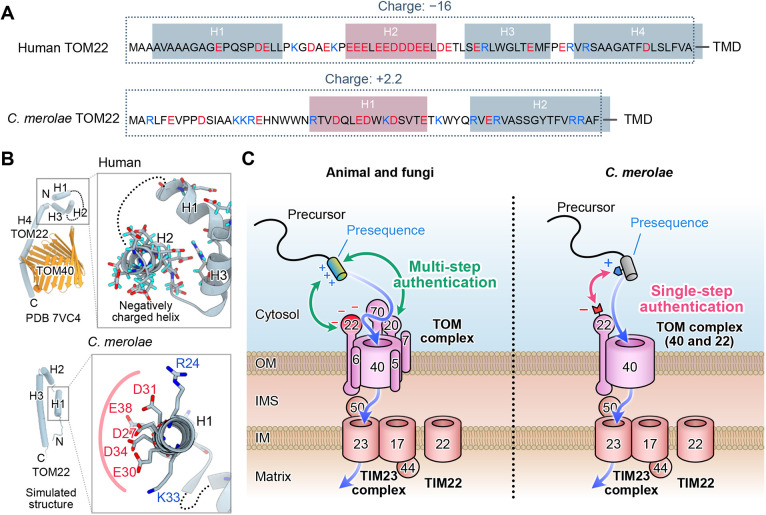
**Schematic representation of mitochondrial protein import in *C. merolae*.** (A) Comparison of the N-terminal domains of human TOM22 and *C. merolae* TOM22. Human TOM22 helix domains and *C. merolae* TOM22 helix domains are shown in boxes. Helical regions for *C. merolae* TOM22 were computationally predicted by NetSurfP2.0. Red boxes indicate negatively charged helices. (B) Protein architectures of TOM22 and TOM40. Human TOM22 and TOM40 are depicted using protein structural data (PDB: 7VC4). The structure for *C. merolae* TOM22 was computationally simulated. Detailed structures of the boxed areas are shown on the right. (C) Schematic models for mitochondrial protein import in animal and fungi (left) and in *C. merolae* (right). In animal and fungi, a mitochondrial presequence, which is an amphiphilic helix, is recognized by TOM20 and TOM22 as a multi-step authentication process. In contrast, a presequence α-helix containing a few basic residues would be recognized by the acidic part of the α-helix in TOM22 via electrostatic residue–residue interaction as a single-step authentication in *C. merolae*. During the process, basic residues on the presequence work as the key and the acidic part on TOM22 functions as the lock. After the authentication, a precursor protein is imported into TOM40 and drawn in by the function of the TIM complex. OM, outer membrane; IMS, intermembrane space; IM, inner membrane.

Interestingly, the N-terminal domains of human TOM22 (142 amino acids) and *C. merolae* TOM22 (119 amino acids), which face toward the cytoplasm *in vivo*, share very low protein sequence identities (Fig. 6A). The estimated charge of the N-terminal domain of human TOM22 is strongly negative (−16) (Fig. 6B, top), suggesting that the N-terminal domain captures the mitochondrial precursors via their positively charged TPs as shown in a recent study (Su et al., 2022). In contrast, the estimated charge of the N-terminal domain of *C. merolae* TOM22 is not negative (+2.2), but we found that one putative α-helical domain has five acidic residues on one side (Fig. 6B, bottom). Thus, in *C. merolae*, mitochondrial precursor proteins would be verified via electrostatic interaction between basic residues on the TP and the acidic residues on TOM22 of the α-helix as the single-step authentication (Fig. 6C). The TOM22-mediated authentication mechanism for mitochondrial protein import would be one reason why the TP with very few basic residues can fulfil the requirement for protein targeting to the mitochondrion in *C. merolae*.

### Diversification of the TOM complex in eukaryotes during evolution

The requirement of arginine residues for protein targeting to mitochondria is also conserved in green plants (Heidorn-Czarna et al., 2022; Lee et al., 2019). However, the protein components of the TOM complex in green plants differ significantly not only from those in fungi/animals, but also from those in red algae (Carrie et al., 2010). In contrast to red algae, no orthologue of TOM22 has been identified in the green plant lineages. Furthermore, although *Arabidopsis thaliana* TOM20 has been shown to be functionally equivalent to animal/fungal TOM20, there is no sequence similarity between *A. thaliana* TOM20 and animal/fungal TOM20 (Perry et al., 2006). Thus, the TOM complex in the green plant lineage is composed of many types of specific proteins, but the authentication mechanism of mitochondrial precursor proteins in green plants could be similar in complexity to those in fungi and animals through convergent evolution. Considering the diversification of the components and the complexity of the structure of TOM complexes in modern eukaryotes, the simple authentication mechanism in the red alga *C. merolae* is very unique and would be a minimal or primitive system.

### Rewriting of the mitochondrial targeting information with an additional polypeptide for targeting to the chloroplast

Our results suggest a putative mechanism for classification of mitochondrial and chloroplast precursor proteins in *C. merolae*, which has the simplest cell structure as a photosynthetic eukaryote. Given that the mean length of the chloroplast TPs is longer than that of the mitochondrial TPs (Fig. 1E,F) and our experimental result for chloroplast RPSA (Fig. 5E), the presence of additional peptide sequences would determine whether the TPs are for the mitochondrion or the chloroplast. The longer length for the chloroplast TP than that for the mitochondrial TP is also found in *A. thaliana*. As the chloroplast arose after the birth of the mitochondrion via endosymbiosis, the protein targeting system for the chloroplast would have to use longer, more complex TPs to distinguish them from mitochondrial TPs.

## MATERIALS AND METHODS

### Cell culture

The *C. merolae* M4 strain, which is isolated from *C. merolae* 10D (NIES-3377) and has a mutation in the *URA5.3* gene, was used in this study (Minoda et al., 2004). The *C. merolae* M4 cells were cultured in MA2 medium (Minoda et al., 2004) supplemented with 0.5 mg/ml uracil and 0.8 mg/ml 5-fluoroorotic acid mono-hydrate in a tissue culture flask 25 (TPP Techno Plastic Products, Switzerland) with shaking at 120 rpm under continuous white light at 40°C.

### Putative presequence dataset

Protein sequences of well-studied mitochondrial and chloroplast proteins were chosen from 4803 *C. merolae* ORFs (Matsuzaki et al., 2004; Mori et al., 2016; Moriyama et al., 2014a,b; Nozaki et al., 2007). By the protein-BLAST search, non-conserved regions at the N-terminal of each protein sequence were identified by visual confirmation and the regions were assessed as putative presequence regions in this study. Through this approach, we created the sequence dataset for 113 mitochondrial presequences (Table S2) and 97 chloroplast presequences (Table S3).

### Data analysis

Prediction of presequences for the mitochondrion and the chloroplast was performed by TargetP 2.0 program (Armenteros et al., 2019) (Table S1). Presequence net charge was calculated for a pH of 7 using the Protein Calculator v3.4 program (https://protcalc.sourceforge.net/). Hydrophobicity and hydrophobic moment of each putative α-helical polypeptide were calculated using the HELIQUEST program (Gautier et al., 2008). Amino acid frequencies were calculated using *C. merolae* ORF data (http://czon.jp/). Sequence logos were made using the WebLogo program (Crooks et al., 2004). Helical wheel maps were made using the NetWheels program (http://lbqp.unb.br/NetWheels/). Protein local structures were predicted by the NetSurfP 2.0 program (Klausen et al., 2019) and tertial structures were simulated by the AlphaFold program (Jumper et al., 2021). Peptide sequence homologies between the synTP and N-terminal 1–24 amino acids of the *C. merolae* ORFs were calculated using BLOSUM30 matrix.

### Transformation and the fluorescent reporter assay

The *C. merolae* cell lines expressing mVenus fused with N-terminal TPs were produced as follows. To generate the presequence-fused mVenus expression vector, a presequence of interest was cloned into the vector using the following methods. To produce the strains expressing AAT (aspartate aminotransferase, CMC148C) 1–33, 3-R, 2-R, 1-R (R13), 0-R, 3-K, FMNL 1–24, FMNL 1–24 (E2A), FMNL 1–24 (E18A), FMNL 1–24 (E2A/E18A) and RPSA (chloroplast r-protein S1, CMM019C) 1–24, the nucleotide sequence of TPs were added to the 5′ end of *mVenus* by two-step PCR and cloned into the linearized pQE80L vector (Qiagen) containing the *URA5.3* upstream sequence (from −2300 to −898 bp), the 500 bp of *CpcC* promoter, the 200 bp of *TUBB* downstream sequence and the *URA5.3* selection marker. To produce the strains 1-R (R2), 1-R (R6), 1-R (R8), 1-R (R9), 1-R (R12), 1-R (R15) and 1-R (R21), synthetic DNA fragments were cloned into the linearized vector.

Using the resulting constructs as a template, DNA fragments for homologous recombination comprising ∼1400 bp of sequence upstream of *URA5.3*, the *CpcC* promoter, the nucleotide sequence of theTP, *mVenus*, the *TUBB* 3′ untranslated region and the *URA5.3* selection marker were amplified by PCR. The PCR amplicons were introduced upstream of the chromosomal *ura5.3* of the uracil auxotrophic mutant strain M4 by polyethylene glycol (PEG)-mediated transformation as described previously (Fujiwara et al., 2015; Imamura et al., 2009; Ohnuma et al., 2008). Positive clones, which are non-uracil-auxotrophic, on the MA2 plate were confirmed by Sanger sequencing and observed by fluorescence microscopy. See also Fig. S3.

All PCR amplifications were performed with Platinum SuperFi II DNA polymerase (Thermo Fisher Scientific). Purification and assembly of DNA fragments were performed using a Wizard SV Gel and PCR Clean-up System (Promega) and a NEBuilder HiFi DNA assembly cloning kit (New England Biolabs). DNA sequences are listed in Table S7.

### Fluorescence microscopy

Fluorescence observations were performed on an Olympus IX83 inverted microscope with a 1.45 NA, 100× oil immersion objective. Illumination was provided by a fluorescent light source (U-HGLGPS; Olympus), excitation filters [490-500HQ (Olympus) for mVenus, FF01-405/10-25 (Semrock) for chloroplasts], custom dichroic mirrors [Di03-R514-t1-25×36 (mVenus), Di03-R514-t1-25×36 (Semrock), T455lp (Chroma) (for chloroplasts)] and emission filters [FF02-531/22-25 (Semrock) (for mVenus), FF02-617/73-25 (for chloroplasts) (Semrock)]. Images were acquired with an ORCA Fusion BT sCMOS camera (Hamamatsu Photonics) controlled by MetaMorph software (Molecular Devices). The effective pixel size was 65.2 nm×65.2 nm.

### Phylogenetic analysis of *C. merolae* RPSA

The amino acid sequences of chloroplast r-protein S1 (CMM019C) and mitochondrial r-protein S1 (CMI304C) of *C. merolae* were retrieved from the *Cyanidioschyzon merolae* Genome Project v3 (Matsuzaki et al., 2004; Nozaki et al., 2007) and used as a query for BLASTP (https://blast.ncbi.nlm.nih.gov/Blast.cgi). The BLASTP was carried out against the non-redundant protein sequences (nr) of all organisms in the National Center for Biotechnology Information (NCBI) database (https://www.ncbi.nlm.nih.gov/). The top 50 sequences with the highest *E*-value sequences of each query were retrieved. We also added amino acid sequences annotated with chloroplast r-protein S1 of six Viridiplantae species from NCBI. Multiple sequence alignments were generated using the MAFFT v7.511 online service in auto strategy (Katoh et al., 2018; Kuraku et al., 2013). Non-homologous regions were detected and cleaned using HMMcleaner (Di Franco et al., 2019). Finally, the multiple sequence alignment was trimmed using trimAl by automated trimming heuristic using the ‘-automated1’ option (Capella-Gutiérrez et al., 2009). Redundant operational taxonomic units (OTUs) that became identical after the trimming procedures were manually excluded. Finally, 83 OTUs were used for analysis. Bayesian inference for the alignments was carried out using MrBayes v3.2.7a MPI version with the best-fitted model selected by ModelTest-NG v0.1.7 (Altekar et al., 2004; Darriba et al., 2020; Flouri et al., 2015). Convergences of Markov chain Monte Carlo iterations were evaluated based on the average standard deviation of split frequencies for every 1,000,000 generations, discarding the first 25% as burn-in, and the iterations were automatically stopped when the average standard deviations were below 0.01, indicating convergence. In addition, the maximum likelihood method was subjected to the alignment with bootstrap values based on 1000 replications by RAxML-NG v1.2.0 with the same model as Bayesian inference (Kozlov et al., 2019). The alignments used for the phylogenetic analysis are uploaded to TreeBASE (Vos et al., 2012).

## Supplementary Material



10.1242/joces.262042_sup1 Supplementary information

Table S1. List of results of the presequence prediction for 4,803 *C. merolae* ORFs.

## References

[JCS262042C1] Abe, Y., Shodai, T., Muto, T., Mihara, K., Torii, H., Nishikawa, S., Endo, T. and Kohda, D. (2000). Structural basis of presequence recognition by the mitochondrial protein import receptor Tom20. *Cell* 100, 551-560. 10.1016/S0092-8674(00)80691-1 10721992

[JCS262042C2] Altekar, G., Dwarkadas, S., Huelsenbeck, J. P. and Ronquist, F. (2004). Parallel Metropolis coupled Markov chain Monte Carlo for Bayesian phylogenetic inference. *Bioinformatics* 20, 407-415. 10.1093/bioinformatics/btg427 14960467

[JCS262042C3] Araiso, Y., Tsutsumi, A., Qiu, J., Imai, K., Shiota, T., Song, J., Lindau, C., Wenz, L. S., Sakaue, H., Yunoki, K. et al. (2019). Structure of the mitochondrial import gate reveals distinct preprotein paths. *Nature* 575, 395-401. 10.1038/s41586-019-1680-7 31600774

[JCS262042C4] Armenteros, J. J. A., Salvatore, M., Emanuelsson, O., Winther, O., Von Heijne, G., Elofsson, A. and Nielsen, H. (2019). Detecting sequence signals in targeting peptides using deep learning. *Life Sci. Alliance* 2, 1-14. 10.26508/lsa.201900429 PMC6769257 31570514

[JCS262042C5] Calvo, S. E., Julien, O., Clauser, K. R., Shen, H., Kamer, K. J., Wells, J. A. and Mootha, V. K. (2017). Comparative analysis of mitochondrial N-termini from mouse, human, and yeast. *Mol. Cell. Proteomics* 16, 512-523. 10.1074/mcp.M116.063818 28122942 PMC5383775

[JCS262042C6] Capella-Gutiérrez, S., Silla-Martínez, J. M. and Gabaldón, T. (2009). trimAl: A tool for automated alignment trimming in large-scale phylogenetic analyses. *Bioinformatics* 25, 1972-1973. 10.1093/bioinformatics/btp348 19505945 PMC2712344

[JCS262042C7] Carrie, C., Murcha, M. W. and Whelan, J. (2010). An *in silico* analysis of the mitochondrial protein import apparatus of plants. *BMC Plant Biol.* 10, 1-15. 10.1186/1471-2229-10-249 21078193 PMC3095331

[JCS262042C8] Carrie, C., Venne, A. S., Zahedi, R. P. and Soll, J. (2015). Identification of cleavage sites and substrate proteins for two mitochondrial intermediate peptidases in *Arabidopsis thaliana*. *J. Exp. Bot.* 66, 2691-2708. 10.1093/jxb/erv064 25732537 PMC4986872

[JCS262042C9] Chacinska, A., Koehler, C. M., Milenkovic, D., Lithgow, T. and Pfanner, N. (2009). Importing mitochondrial proteins: Machineries and mechanisms. *Cell* 138, 628-644. 10.1016/j.cell.2009.08.005 19703392 PMC4099469

[JCS262042C10] Crooks, G., Hon, G., Chandonia, J. and Brenner, S. (2004). WebLogo: a sequence logo generator. *Genome Res.* 14, 1188-1190. 10.1101/gr.849004 15173120 PMC419797

[JCS262042C11] Darriba, D., Posada, D., Kozlov, A. M., Stamatakis, A., Morel, B. and Flouri, T. (2020). ModelTest-NG: A new and scalable tool for the selection of DNA and protein evolutionary models. *Mol. Biol. Evol.* 37, 291-294. 10.1093/molbev/msz189 31432070 PMC6984357

[JCS262042C12] Di Franco, A., Poujol, R., Baurain, D. and Philippe, H. (2019). Evaluating the usefulness of alignment filtering methods to reduce the impact of errors on evolutionary inferences. *BMC Evol. Biol.* 19, 1-17. 10.1186/s12862-019-1350-2 30634908 PMC6330419

[JCS262042C13] Endo, T. and Yamano, K. (2009). Multiple pathways for mitochondrial protein traffic. *Biol. Chem.* 390, 723-730. 10.1515/BC.2009.087 19453276

[JCS262042C14] Flouri, T., Izquierdo-Carrasco, F., Darriba, D., Aberer, A. J., Nguyen, L. T., Minh, B. Q., Von Haeseler, A. and Stamatakis, A. (2015). The phylogenetic likelihood library. *Syst. Biol.* 64, 356-362. 10.1093/sysbio/syu084 25358969 PMC4380035

[JCS262042C15] Fujiwara, T., Kanesaki, Y., Hirooka, S., Era, A., Sumiya, N., Yoshikawa, H., Tanaka, K. and Miyagishima, S. Y. (2015). A nitrogen source-dependent inducible and repressible gene expression system in the red alga *Cyanidioschyzon merolae*. *Front. Plant Sci.* 6, 1-10. 10.3389/fpls.2015.00657 26379685 PMC4549557

[JCS262042C16] Garg, S. G. and Gould, S. B. (2016). The role of charge in protein targeting evolution. *Trends Cell Biol.* 26, 894-905. 10.1016/j.tcb.2016.07.001 27524662

[JCS262042C17] Gautier, R., Douguet, D., Antonny, B. and Drin, G. (2008). HELIQUEST: A web server to screen sequences with specific α-helical properties. *Bioinformatics* 24, 2101-2102. 10.1093/bioinformatics/btn392 18662927

[JCS262042C18] Gavel, Y., Nilsson, L. and von Heijne, G. (1988). Mitochondrial targeting sequences why “non-amphiphilic” peptides may still be amphiphilic. *FEBS Lett.* 235, 173-177. 10.1016/0014-5793(88)81257-2 3402595

[JCS262042C19] Gray, M. W., Burger, G. and Lang, B. F. (1999). Mitochondrial Evolution. *Science* 283, 1476-1481. 10.1126/science.283.5407.1476 10066161

[JCS262042C20] Hanif Sayyed, U. M. and Mahalakshmi, R. (2022). Mitochondrial protein translocation machinery: From TOM structural biogenesis to functional regulation. *J. Biol. Chem.* 298, 101870. 10.1016/j.jbc.2022.101870 35346689 PMC9052162

[JCS262042C21] Heidorn-Czarna, M., Maziak, A. and Janska, H. (2022). Protein processing in plant mitochondria compared to yeast and mammals. *Front. Plant Sci.* 13, 1-20. 10.3389/fpls.2022.824080 PMC8847149 35185991

[JCS262042C22] Huang, S., Taylor, N. L., Whelan, J. and Millar, A. H. (2009). Refining the definition of plant mitochondrial presequences through analysis of sorting signals, N-terminal modifications, and cleavage motifs. *Plant Physiol.* 150, 1272-1285. 10.1104/pp.109.137885 19474214 PMC2705053

[JCS262042C23] Imai, K. and Nakai, K. (2020). Tools for the recognition of sorting signals and the prediction of subcellular localization of proteins from their amino acid sequences. *Front. Genet.* 11, 1-12. 10.3389/fgene.2020.607812 33324450 PMC7723863

[JCS262042C24] Imamura, S., Kanesaki, Y., Ohnuma, M., Inouye, T., Sekine, Y., Fujiwara, T., Kuroiwa, T. and Tanaka, K. (2009). R2R3-type MYB transcription factor, CmMYB1, is a central nitrogen assimilation regulator in *Cyanidioschyzon merolae*. *Proc. Natl. Acad. Sci. USA* 106, 14180. 10.1073/pnas.0902790106 19592510 PMC2718362

[JCS262042C25] Jumper, J., Evans, R., Pritzel, A., Green, T., Figurnov, M., Ronneberger, O., Tunyasuvunakool, K., Bates, R., Žídek, A., Potapenko, A. et al. (2021). Highly accurate protein structure prediction with AlphaFold. *Nature* 596, 583-589. 10.1038/s41586-021-03819-2 34265844 PMC8371605

[JCS262042C26] Katoh, K., Rozewicki, J. and Yamada, K. D. (2018). MAFFT online service: Multiple sequence alignment, interactive sequence choice and visualization. *Brief. Bioinform* 20, 1160-1166. 10.1093/bib/bbx108 PMC6781576 28968734

[JCS262042C27] Klausen, M. S., Jespersen, M. C., Nielsen, H., Jensen, K. K., Jurtz, V. I., Sønderby, C. K., Sommer, M. O. A., Winther, O., Nielsen, M., Petersen, B. et al. (2019). NetSurfP-2.0: Improved prediction of protein structural features by integrated deep learning. *Proteins Struct. Funct. Bioinforma* 87, 520-527. 10.1002/prot.25674 30785653

[JCS262042C28] Kozlov, A. M., Darriba, D., Flouri, T., Morel, B. and Stamatakis, A. (2019). RAxML-NG: A fast, scalable and user-friendly tool for maximum likelihood phylogenetic inference. *Bioinformatics* 35, 4453-4455. 10.1093/bioinformatics/btz305 31070718 PMC6821337

[JCS262042C29] Kuraku, S., Zmasek, C. M., Nishimura, O. and Katoh, K. (2013). aLeaves facilitates on-demand exploration of metazoan gene family trees on MAFFT sequence alignment server with enhanced interactivity. *Nucleic Acids Res.* 41, 22-28. 10.1093/nar/gkt389 PMC3692103 23677614

[JCS262042C30] Kuroiwa, T. (1998). The primitive red algae *Cyanidium caldarium* and *Cyanidioschyzon merolae* as model system for investigating the dividing apparatus of mitochondria and plastids. *BioEssays* 20, 344-354. 10.1002/(SICI)1521-1878(199804)20:4<344::AID-BIES11>3.0.CO;2-2

[JCS262042C31] Lee, D. W., Lee, S., Lee, J., Woo, S., Razzak, M. A., Vitale, A. and Hwang, I. (2019). Molecular mechanism of the specificity of protein import into chloroplasts and mitochondria in plant cells. *Mol. Plant* 12, 951-966. 10.1016/j.molp.2019.03.003 30890495

[JCS262042C32] Matsuzaki, M., Misumi, O., Shin-i, T., Maruyama, S., Takahara, M., Miyagishima, S.-Y., Mori, T., Nishida, K., Yagisawa, F., Nishida, K. et al. (2004). Genome sequence of the ultrasmall unicellular red alga *Cyanidioschyzon merolae* 10D. *Nature* 428, 653-657. 10.1038/nature02398 15071595

[JCS262042C33] Minoda, A., Sakagami, R., Yagisawa, F., Kuroiwa, T. and Tanaka, K. (2004). Improvement of culture conditions and evidence for nuclear transformation by homologous recombination in a red alga, *Cyanidioschyzon merolae* 10D. *Plant Cell Physiol.* 45, 667-671. 10.1093/pcp/pch087 15215501

[JCS262042C34] Mori, N., Moriyama, T., Toyoshima, M. and Sato, N. (2016). Construction of global acyl lipid metabolic map by comparative genomics and subcellular localization analysis in the red alga *Cyanidioschyzon merolae*. *Front. Plant Sci.* 7, 1-14. 10.3389/fpls.2016.00958 27446184 PMC4928187

[JCS262042C35] Moriyama, T., Sakurai, K., Sekine, K. and Sato, N. (2014a). Subcellular distribution of central carbohydrate metabolism pathways in the red alga *Cyanidioschyzon merolae*. *Planta* 240, 585-598. 10.1007/s00425-014-2108-0 25009310

[JCS262042C36] Moriyama, T., Tajima, N., Sekine, K. and Sato, N. (2014b). Localization and phylogenetic analysis of enzymes related to organellar genome replication in the unicellular rhodophyte *Cyanidioschyzon merolae*. *Genome Biol. Evol.* 6, 228-237. 10.1093/gbe/evu009 24407855 PMC3914683

[JCS262042C37] Nagai, T., Ibata, K., Park, E. S., Kubota, M., Mikoshiba, K. and Miyawaki, A. (2002). A variant of yellow fluorescent protein with fast and efficient maturation for cell-biological applications. *Nat. Biotechnol.* 20, 87-90. 10.1038/nbt0102-87 11753368

[JCS262042C38] Nargang, F. E., Rapaport, D., Ritzel, R. G., Neupert, W. and Lill, R. (1998). Role of the negative charges in the cytosolic domain of TOM22 in the import of precursor proteins into mitochondria. *Mol. Cell. Biol.* 18, 3173-3181. 10.1128/MCB.18.6.3173 9584158 PMC108899

[JCS262042C39] Nozaki, H., Takano, H., Misumi, O., Terasawa, K., Matsuzaki, M., Maruyama, S., Nishida, K., Yagisawa, F., Yoshida, Y., Fujiwara, T. et al. (2007). A 100%-complete sequence reveals unusually simple genomic features in the hot-spring red alga *Cyanidioschyzon merolae*. *BMC Biol.* 5, 1-8. 10.1186/1741-7007-5-28 17623057 PMC1955436

[JCS262042C40] Ohnuma, M., Yokoyama, T., Inouye, T., Sekine, Y. and Tanaka, K. (2008). Polyethylene glycol (PEG)-mediated transient gene expression in a red alga, *Cyanidioschyzon merolae* 10D. *Plant Cell Physiol.* 49, 117-120. 10.1093/pcp/pcm157 18003671

[JCS262042C41] Pace, C. N. and Scholtz, J. M. (1998). A helix propensity scale based on experimental studies of peptides and proteins. *Biophys. J.* 75, 422-427. 10.1016/S0006-3495(98)77529-0 9649402 PMC1299714

[JCS262042C42] Perry, A. J., Hulett, J. M., Likić, V. A., Lithgow, T. and Gooley, P. R. (2006). Convergent evolution of receptors for protein import into mitochondria. *Curr. Biol.* 16, 221-229. 10.1016/j.cub.2005.12.034 16461275

[JCS262042C43] Pfanner, N., Warscheid, B. and Wiedemann, N. (2019). Mitochondrial proteins: from biogenesis to functional networks. *Nat. Rev. Mol. Cell Biol.* 20, 267-284. 10.1038/s41580-018-0092-0 30626975 PMC6684368

[JCS262042C44] Saitoh, T., Igura, M., Obita, T., Ose, T., Kojima, R., Maenaka, K., Endo, T. and Kohda, D. (2007). Tom20 recognizes mitochondrial presequences through dynamic equilibrium among multiple bound states. *EMBO J.* 26, 4777-4787. 10.1038/sj.emboj.7601888 17948058 PMC2080804

[JCS262042C45] Schmidt, O., Pfanner, N. and Meisinger, C. (2010). Mitochondrial protein import: From proteomics to functional mechanisms. *Nat. Rev. Mol. Cell Biol.* 11, 655-667. 10.1038/nrm2959 20729931

[JCS262042C46] Shiota, T., Mabuchi, H., Tanaka-Yamano, S., Yamano, K. and Endo, T. (2011). In vivo protein-interaction mapping of a mitochondrial translocator protein Tom22 at work. *Proc. Natl. Acad. Sci. USA* 108, 15179-15183. 10.1073/pnas.1105921108 21896724 PMC3174609

[JCS262042C47] Shiota, T., Imai, K., Qiu, J., Hewitt, V. L., Tan, K., Shen, H. H., Sakiyama, N., Fukasawa, Y., Hayat, S., Kamiya, M. et al. (2015). Molecular architecture of the active mitochondrial protein gate. *Science* 349, 1544-1548. 10.1126/science.aac6428 26404837

[JCS262042C48] Sickmann, A., Reinders, J., Wagner, Y., Joppich, C., Zahedi, R., Meyer, H. E., Schönfisch, B., Perschil, I., Chacinska, A., Guiard, B. et al. (2003). The proteome of *Saccharomyces cerevisiae* mitochondria. *Proc. Natl. Acad. Sci. USA* 100, 13207-13212. 10.1073/pnas.2135385100 14576278 PMC263752

[JCS262042C49] Sim, S. I., Chen, Y., Lynch, D. L., Gumbart, J. C. and Park, E. (2023). Structural basis of mitochondrial protein import by the TIM23 complex. *Nature* 621, 620-626. 10.1038/s41586-023-06239-6 37344598 PMC11495887

[JCS262042C50] Su, J., Liu, D., Yang, F., Zuo, M. Q., Li, C., Dong, M. Q., Sun, S. and Sui, S. F. (2022). Structural basis of Tom20 and Tom22 cytosolic domains as the human TOM complex receptors. *Proc. Natl. Acad. Sci. USA* 119, 1-10. 10.1073/pnas.2200158119 PMC9245660 35733257

[JCS262042C51] Tanaka, N., Mogi, Y., Fujiwara, T., Yabe, K., Toyama, Y., Higashiyama, T. and Yoshida, Y. (2021). CZON-cutter – a CRISPR-Cas9 system for multiplexed organelle imaging in a simple unicellular alga. *J. Cell Sci.* 134, 1-11. 10.1242/jcs.258948 34633046

[JCS262042C52] Vögtle, F. N., Wortelkamp, S., Zahedi, R. P., Becker, D., Leidhold, C., Gevaert, K., Kellermann, J., Voos, W., Sickmann, A., Pfanner, N. et al. (2009). Global analysis of the mitochondrial N-proteome identifies a processing peptidase critical for protein stability. *Cell* 139, 428-439. 10.1016/j.cell.2009.07.045 19837041

[JCS262042C53] von Heijne, G., Steppuhn, J. and Herrmann, R. G. (1989). Domain structure of mitochondrial and chloroplast targeting peptides. *Eur. J. Biochem.* 180, 535-545. 10.1111/j.1432-1033.1989.tb14679.x 2653818

[JCS262042C54] Vos, R. A., Balhoff, J. P., Caravas, J. A., Holder, M. T., Lapp, H., Maddison, W. P., Midford, P. E., Priyam, A., Sukumaran, J., Xia, X. et al. (2012). NeXML: Rich, extensible, and verifiable representation of comparative data and metadata. *Syst. Biol.* 61, 675-689. 10.1093/sysbio/sys025 22357728 PMC3376374

[JCS262042C55] Yamamoto, H., Esaki, M., Kanamori, T., Tamura, Y., Nishikawa, S. and Endo, T. (2002). Tim50 is a subunit of the TIM23 complex that links protein translocation across the outer and inner mitochondrial membranes. *Cell* 111, 519-528. 10.1016/S0092-8674(02)01053-X 12437925

[JCS262042C56] Zhou, X., Yang, Y., Wang, G., Wang, S., Sun, D., Ou, X., Lian, Y. and Li, L. (2023). Molecular pathway of mitochondrial preprotein import through the TOM–TIM23 supercomplex. *Nat. Struct. Mol. Biol.* 30, 1996-2008. 10.1038/s41594-023-01103-7 37696957

