# Supplementary material for: Simple prerequisite of presequence for mitochondrial protein import in the unicellular red alga Cyanidioschyzon merolae
Source: J Cell Sci. 2024 Jul 23;137(14):jcs262042. doi: 10.1242/jcs.262042 (PMC11298712; doi:10.1242/jcs.262042)
Supplement: Supplementary information [file joces-137-262042-s1.pdf]

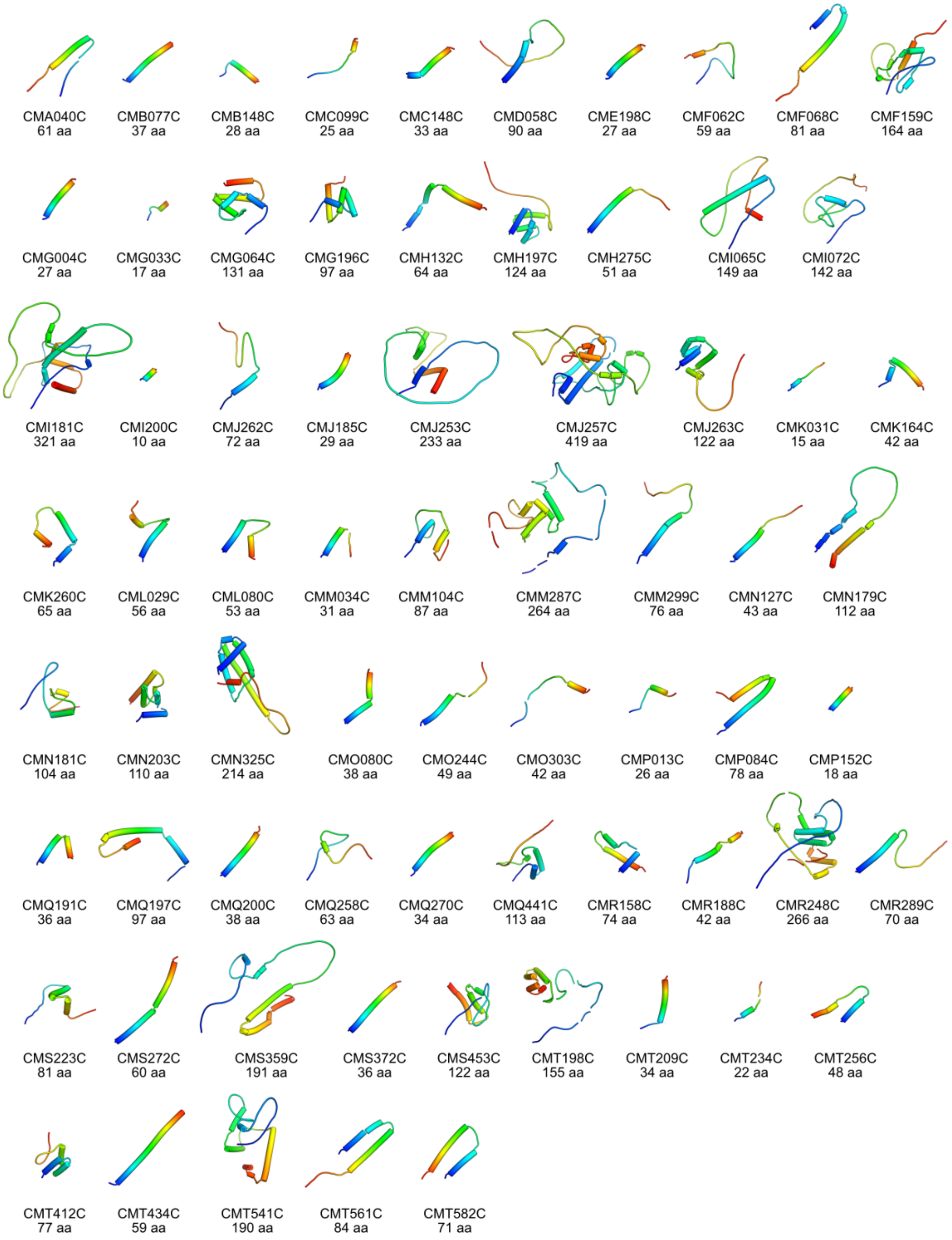

Fig. S1. Simulated structures of mitochondrial presequences containing α-helical regions. In the 113 mitochondrial presequences, 71 presequences contain α-helical structures as a secondary structure. Tertial protein models of mitochondrial presequences are rainbow-colored from the N (blue) to the C terminus (red).

Journal of Cell Science • Supplementary information

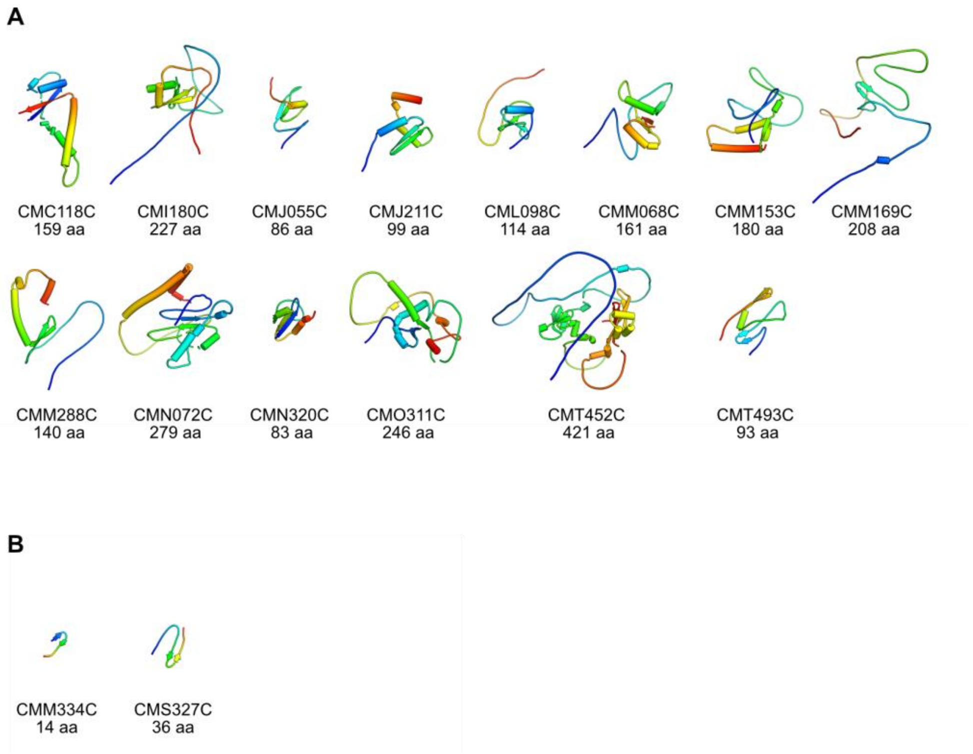

Fig. S2. Simulated structures of mitochondrial presequences containing α-helical and β-sheet regions. (**A** and **B**) Presequences containing both α-helix and β-sheet regions (**A**) and only β-sheet regions (**B**). In the 113 mitochondrial presequences, 14 presequences contain both α-helical and β-sheet structures and 2 presequences contain only β-sheet structures. Tertial protein models of mitochondrial presequences are rainbow-colored from the N (blue) to the C terminus (red).

Journal of Cell Science • Supplementary information

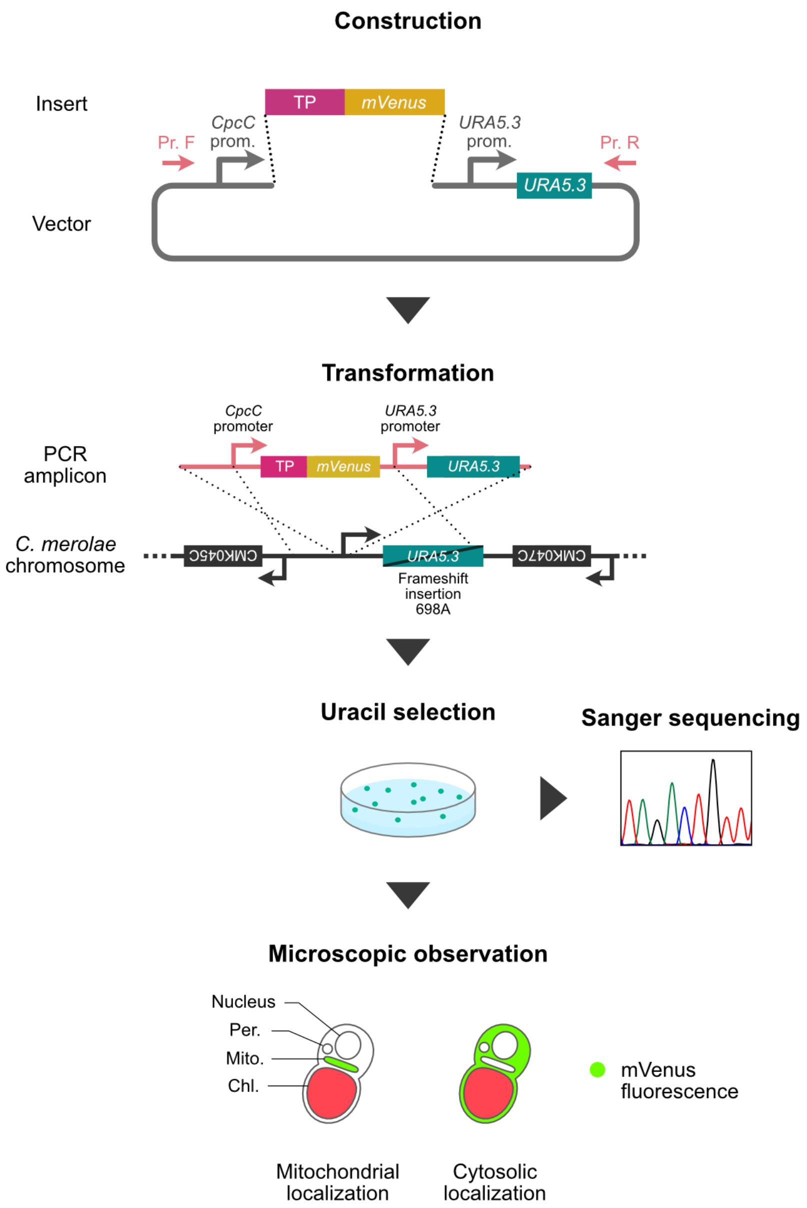

Fig. S3. Schematic representation of the fluorescent reporter assay for evaluation of the protein targeting to the mitochondrion. Further details on the construction of the plasmids and fluorescence microscopy are described in the Materials and Methods section.

Journal of Cell Science • Supplementary information

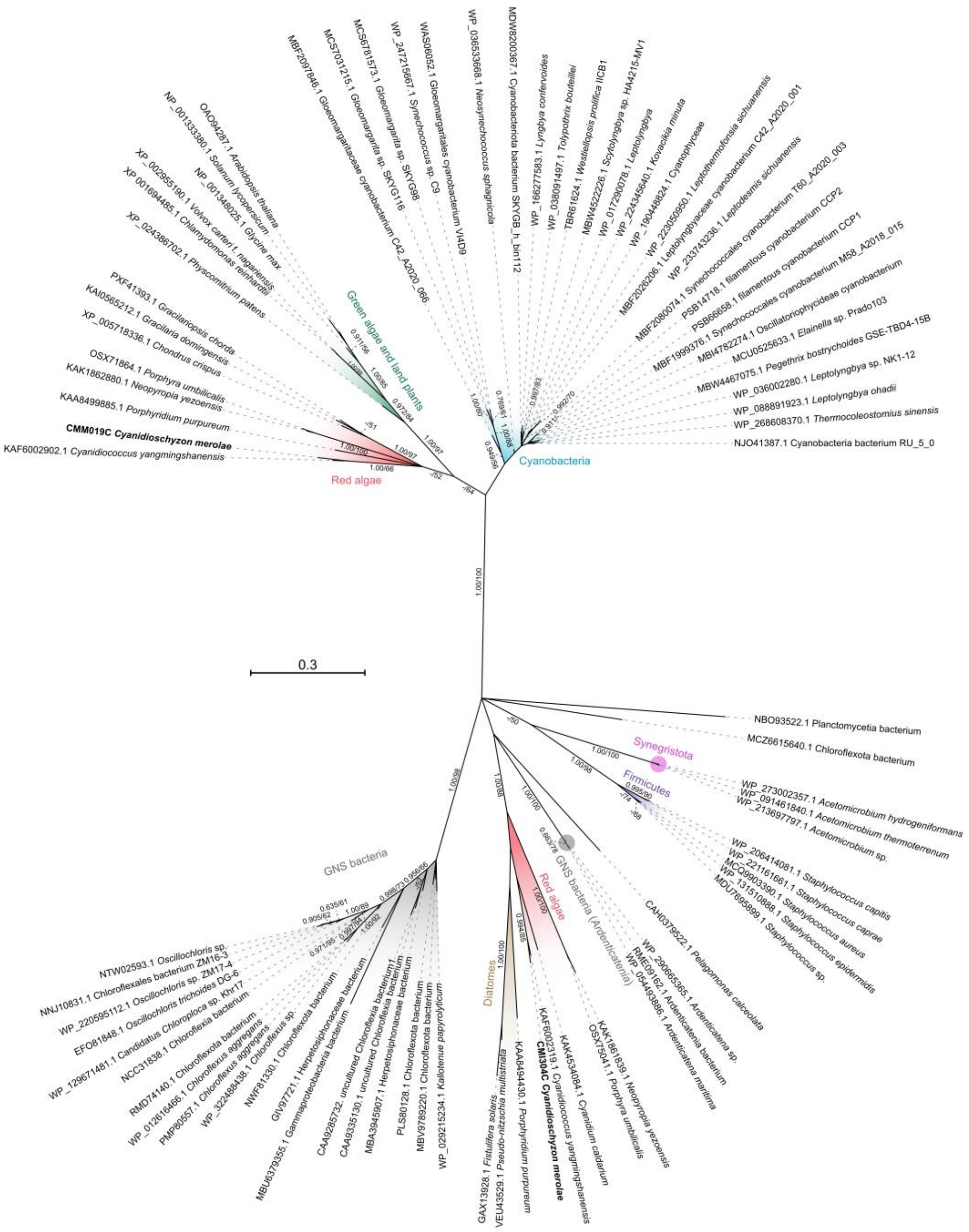

Fig. S4. The unabbreviated Bayesian tree of chloroplast, mitochondrial and bacterial RPSA proteins. Phylogenetic analysis of *C. merolae* chloroplast and mitochondrial RPSAs (CMM019C and CMI304C), inferred based on 226 amino acid sequences by the Bayesian inference (BI) using the LG + G4 model. The number before the species name of each OTU indicates the NCBI accession number for the sequence. Branch lengths are proportional to the evolutionary distances indicated by the scale bar. Numbers at left and right above branches indicate posterior probabilities of BI (≥0.90) and bootstrap values of the maximum likelihood method (≥50%), respectively.

Table S1. List of results of the presequence prediction for 4,803 *C. merolae* ORFs.

Available for download at
https://journals.biologists.com/jcs/article-lookup/doi/10.1242/jcs.262042#supplementary-data

Table S2. List of 113 putative mitochondrial TP sequences.

#	Gene ID	Estimated mTP sequence	Length	Length (TargetP2.0)	Score (TargetP2.0)	Annotation
1	CMA040C	MVSQKETSRADEVTITIPVEPGDAEAGRAAAVSAAAVGRALQTALQRKASAAPRLAPREVW	61	ND	0.00	citrate synthase
2	CMA090C	MSGSSNGRLQASEAPIAK	18	ND	0.00	NADH dehydrogenase I (Complex I) alpha subcomplex 8 (PGIV)
3	CMB077C	MSLKGVAFFLLKGVAELSKNATSLLKLASAVYFFQKN	37	ND	0.15	similar to mitochondrial inner membrane protease IMP1
4	CMB148C	MDKIDQLTPSQRAELEDRVRNEMLRQAF	28	ND	0.00	probable mitochondrial intermembrane space complex subunit Tim13
5	CMC099C	MTGRHVESVDTDPKPVDEAFCIANK	25	ND	0.00	NADH dehydrogenase I (Complex I) beta subcomplex 7 (B18)
6	CMC118C	MLRQCLFGVGRLLSGEQGLKNASRFLARGIHARAAHALPVTRASTPGATLVQRDPFRRLDCIDLGKLQVRRLQSESSRAVGRAESATAVLGAESPKSTGPGTTTVDSEATAKIADDLRASSNAASTDLASEAAEAALSAERNWYDPVVQGIIMFQEYTD	159	43	0.93	similar to oxidase (cytochrome c) assembly OXA1, mitochondrial precursor
7	CMC148C	MFVGLLTRRNLRPLQSLARKQSQLDTFRSRMSY	33	33	0.99	aspartate aminotransferase
8	CMD058C	MSAQRARAVSSLWASLRPLEAYLAAGEYQGGPVFQRRPASTGIRGGTSHNAEHALPGGGVRSALQTTPPVASYTRCFGSSVERPAMQGDT	90	77	0.62	fumarate hydratase precursor
9	CME198C	MVRSNLGLLASCGAAYWRRLATSAGSA	27	20	0.68	mitochondrial F1FO ATP synthase subunit ATP5
10	CMF062C	MASTFKTTAGTGAGEETHARDSYPGRDTTIRREELPSGLRDALPYTSSSWWWRAIERTE	59	ND	0.00	mitochondrial general import pore subunit Tom40
11	CMF068C	MLRVVRALNPILFRSLSPTGVSKRLLGLTGCNVLGARLLSTASASSSSDAATGAAAAAAAATAAQTTHRQPTLAESGLSSA	81	39	0.97	2-oxoglutarate dehydrogenase, E1 component
12	CMF129C	MSALS	5	12	0.52	mitochondrial chaperonin hsp10, precursor
13	CMF159C	MQALCSRRLLVHLWRGSALSGAQKAAVHLRCSGADSQNAGLLRREVVHRVVRAPDELLARCLSANRWNSSFRDLYPRGWKKGKPPEEQPTLKPGVGSPADREGLASDTQPERGDASRAKGSRSGGGAPGPNWWALLVFGSGLAAALWLSGSESGASNPFSTPKP	164	31	0.92	AAA-metalloprotease FtsH, mitochondrial precursor
14	CMG004C	MVRSNLGLLARYGAAYWRRLATSAGSA	27	20	0.80	mitochondrial F1FO ATP synthase subunit ATP5
15	CMG033C	MASALAAGVNPVDLARR	17	ND	0.00	mitochondrial intermembrane space complex subunit Tim10
16	CMG064C	MRVVSFPRSSGSALRKRTEAFAGLLSYPWCQHSALELYSRQKVLNTRRLSFLDSGMCLFAGVRTFRSNFLASELRLARATHLNLDLWRRNSSLLRKTHVQYRQASGRGRGPSTADSSDVRKRNLNFLAWSL	131	ND	0.21	cytochrome c oxidase (Complex IV) assembly protein
17	CMG067C	MEADTVAERSEATPAPPLHT	20	ND	0.00	similar to mitochondrial carrier Yel006N
18	CMG196C	MRTFRAFTLQSRLASAAHWPPRPVPAPADDLLRRCDASLMQRLPSAASTRHLLRFQHSEAVSPATDPEQNDRAVQVALEEAQIAARERGLTGAPVGR	97	55	0.86	mitochondrial translation elongation factor G1 (EF-G1)
19	CMG215C	MSET	4	ND	0.00	cytochrome c reductase (Complex III) hinge protein
20	CMH075C	MSSADSREHKRLA	13	ND	0.01	similar to mitochondrial carrier protein
21	CMH132C	MHALRLLHGKLSVYGQSPCSMLRFLERGTRRWAQLPVSGLAVYRTLTHQARSTKQNTEQVFRGK	64	52	0.95	succinyl-CoA synthetase alpha chain
22	CMH173C	MQSTVPSTKAPEASNGTVARQSTEAVHQQRSKPGLFAQLAV	41	ND	0.00	similar to mitochondrial uncoupling protein
23	CMH197C	MRCVRSIGRTASALARSGKTTFSSTWTSCSKVASIGGCKISTSVPRAVHGVDCFGRFAAVPASLTSLLIGYGARTLATKAGSTTASSASATEVASAGTGKASSASAEHSATSKRPGSPATAVSR	124	76	0.95	mitochondrial F-type ATPase F1 subunit beta, precursor
24	CMH275C	MAASLLHTWKRYLCSSCLNQALAASRRCLRLARYQLPPNGFPRRGSASKAG	51	45	0.52	mitochondrial ribosomal protein L46
25	CMI065C	MFLRLHQRWPWRNYGPWRGRWLLPARDAGTPACRKQVSVVEQSRQSVLDWRFNRLLQAGFYGLGSLLAYGRAPAVRWTSSGSVPQREAPIIAKTLQPVQPTGTTADAGKSTSEGEPWASLGSSSAPTAELLALEIRAESPHAFIQRRLA	149	77	0.62	similar to mitochondrial magnesium transporter Mrs2p
26	CMI072C	MVCAGIVPKVTSIYCRVVGRAVRATGVGDLLARQQGLWRNVPGNAPHLPCRDWSLAGCLSSKSLTTWSATYTARGVRESEPRLPPVGLAAAPLWRTGARALSTQPQPDTSSTTTITDATGRPDETPYQPVRDAPPKSDPNYP	142	ND	0.27	mitochondrial alternative oxidase

Journal of Cell Science • Supplementary information

27	CMI095C	MSQVFILKRVFSRCTRPLLPVVERNSG AASCFVTGYHGAALSTQADTVPSVRP GTAPRVLKSSAPSLPVGMHPEGKL	77	41	0.89	NADH dehydrogenase I (Complex I) iron-sulfur protein 20kDa subunit (PSST)
28	CMI180C	MRERLPSPGRSGTPALQSTRGHDSLQ RLRVRPASFSQTSSAADVPTSPLVLPP LVGGMQQGVSDFGRASTGRGPLSSD APPPSNRTVGIGGSEGVQKGDDEGQD GFFIGNNLFLAKGLTTAAALADEQLTG AAPSRLGPSGNGENNPLTLRRTLSCIEI DPFGKAQVRVYTREELIRELRSESVTG NTAVDVVELAFIRGDAREESQMSGAL DLAPVTGGNRRTKSS	227	ND	0.05	similar to mitochondrial magnesium transporter Mrs2p
29	CMI181C	MEASDSESDGGVPILPLNASEGLDDET ILHPAPPCSESEANARESAVTVAVAGS SPLLETGPPPVAAEPFEPAPESGLYSS KETNFRRSLSSGALEGLRSRNQTLELF QQSIRNQLLLQRATQRATPDSLHTRER PDNAEPSSSSGPEHETNLHGALTTNE RDLYRDSGRAEDSPVSERRQGALPSS AENVSPEHRDSHDEEFPQPPTLRPSG RARGQAPEPIATTPYAGFGQHTPAEQ KKPKRALNTLLCFRFGFNGHATVEKIT TTEILAAARANDQIRAPSSANCIPADSQ SEETLHHAVWRQRKKERRKLQAELRG I	321	ND	0.00	similar to mitochondrial magnesium transporter Mrs2p
30	CMI200C	MAWVARLSHV	10	ND	0.34	NADH dehydrogenase I (Complex I) alpha subcomplex 2 (B8)
31	CMI216C	MSFGSSSSSGFGDLSDSPSSPAQQ	24	ND	0.00	probable mitochondrial intermembrane space complex subunit Tim8
32	CMI262C	MQAHVRPSLNRLQKGAAAGLRSAASP SLCRAAATSSVTPDGSDPVLVVGARG VNRPSYTETGASGRDATVDA	72	32	0.62	NADH dehydrogenase I (Complex I) alpha subcomplex 6 (B14)
33	CMJ055C	MIPHVRSVLGRLGRQVRAPLGLSARP VRVWHRDPRIPSWSHRYICWVGALAC SEQRLQRPLGSLLQKMCKRGLSMPSA TRSSPLET	86	80	0.54	2-oxoglutarate dehydrogenase E2 component (dihydrolipoamide succinyltransferase)
34	CMJ185C	MQGAVRGSLQRVVHKRTRWLVGVFT TLRS	29	29	0.96	NADH dehydrogenase I (Complex I) flavoprotein 51kDa subunit
35	CMJ211C	MWCRGFCSGLGLARNLLLHQLGRRYA DPAPHNGFLVRRLLGTGVRALCVDAT RTRPEPGALQPKDYVDLESYNAFSGE VDVQQLEPHEKERMRANLENA	99	25	0.99	NADH dehydrogenase I (Complex I) iron-sulfur protein 49kDa subunit
36	CMJ238C	MSVVFTSTRPSKGAAAPVIDSSLGLRA PVPTVVPFRKLPRAA	42	ND	0.29	mitochondrial ribosomal protein L28 precursor
37	CMJ253C	MWRPITSRQLSVLCWRLGSTALTRTS AARVNAGRRRPNELRPLLRRPCTSET STSAKPVSATARTSAAPEPRTTESAST AEVDQHTSSSSVAEAAPEAVQAYRPG DRRPGWAGFFRGLFGGTQAAIEDTLA AEARRRGQLNDAEPVRASVQRAHRS RSAATSHATSTASAANREAGTALVPQ GRVRSHDDDAQGDASTTTGYASVLNR IFSRFAGSPFMRSVLEAKERVSERL	233	48	0.54	similar to mitochondrial presequence translocase subunit Tim44
38	CMJ257C	MSERLRLLGRRVTLMDVLVQFTSRGR VPPAAINTVRRKVAHLGTLALEYIGDQV ARTKRWLRLSESLRDPAQPSCAGSRV WPSQGSTSTTETIYAEHARLRLIQDSK FAASGTTANRGTALTGSRLARLERTPT SQVRVASTYVARNRRNAQVGGRCSPL ARQAESTGLLYLNPTVARCRVLSVDG GAASLVSEAGSAAEETLPGTGRQVSQ ADDSSCDLRCPETPACAEEAQQRGPG REAKHLASPTSAAPLAPQGERLSSENV REASAWSRAPERISEPASPELGLRGH NEAHGPAVCSLGGNASKRSTPSKPIA QESLFPVADGRPMRSSREPALIKTTGE PLSTQSAKEVANDEKLDWVELQQRIAS DSGLYGNRHALGLERYLNAAELAEAT RLGLPGTMRSSDGYLHDEMDYL	419	ND	0.14	single subunit mitochondrial RNA polymerase
39	CMJ263C	MLSVKRLQTVARISCVLCVDCRSDAAR TVLTSAGAHVLRSCARSTLYQQRCAY HPRMQDAITARAVAFPLYNAGSVSEFL DNERILGLGSDDAHPVQTATQPSSPV GALGRAQRHPQRLHTR	122	ND	0.36	acyl-Coenzyme A thioesterase, mitochondrial precursor
40	CMJ293C	MGDERSASLTVRVSVKNGTQSPDEAR ECEVLESATEPTIAIAARDVALTVQEAT GTHTVC	60	ND	0.00	citrate synthase
41	CMK031C	MGRLADLVLSVPGTR	15	ND	0.07	cytochrome c reductase (Complex III) subunit 7(VI)
42	CMK164C	MHSRGEEEYGALGVEKNPKKTLFFKR FNSVAKHARQFSYSFT	42	ND	0.00	similar to mitochondrial carrier protein
43	CMK260C	MLSFASRGIAEAAWPSGRLRNSIAASL RGARFSTLKATSPSSESGAESGRPPW VWRKLQGWFGFE	65	32	0.84	probable mitochondrial processing peptidase alpha subunit
44	CML029C	MQGVRAVGLSSVRRVYAAARAHVQS STPGGGGGGGGGVGNFLSSSNEKAL GGVRFA	56	22	0.55	mitochondrial F-type ATPase F1 subunit gamma, precursor
45	CML080C	MLRVFRLRLFGWGHFQRRLDRSVPRY SVEVYGQTRSLATPAGVAGAQTAAHL S	53	26	0.97	acyl-CoA dehydrogenase, mitochondrial precursor

Journal of Cell Science • Supplementary information

46	CML098C	MQRFLVCGGLDALLRRTLARATSTSTR WRNLEILDVPVHKDKTFPQGYWFWW RSSGLLVPGGRTRRSALQPSVPCCASI ATKASGSSASLSPPRASAAMDTSNDE ATDLQPPVR	114	ND	0.43	cytochrome c oxidase (Complex IV) subunit Vb
47	CMM030C	MGAAVSMPAQRAAPFAPGLT	20	ND	0.01	NADH dehydrogenase I (Complex I) beta subcomplex 9 (B22)
48	CMM034C	MPALARTVLRTALSKVLHLHRERPLQH CRRF	31	31	0.94	NADH dehydrogenase I (Complex I) iron-sulfur protein 75kDa subunit
49	CMM068C	MPLTKRSARKWNYGHVSVAKEQSLTL NKPGSIRHRTFDKLSEFHLGWLSRSSR QTKQNAARHQPDAAAKRDRACAAAD VVASPKHIARLWLVACRALVPGEAGSH PMEEYRLPDVSLVVESIHSNEKKTHEV ELYANWKASTEPPNVGRVLVVPSARC PYR	161	ND	0.01	citrate synthase
50	CMM104C	MSEWLLPVRASWIGLTRRCFAFQRLG SVRYAHTGSGSSVSSHSTAAAMPLPD PLQTSFQPTGLKRLVKQVFGENAAPLP PGYRVEPR	87	30	0.86	mitochondrial processing peptidase beta subunit (Complex III core subunit 1), precursor
51	CMM153C	MRVLERLFWRSERAGRPQGSSLDQV PGNESQALGDLSGELEHPRSGFRDEL AVRDSTHAHSDNSGGTGTDRAIPALS KRLSGGGASGESTTMVRAFPEHQRLK AFQELECLAFDEKGNFERRAVSRLELL RAARSVQSALIDYPPLLSDSGDDLSQE QIAKLMKRRQKRIHEYRQNFLQI	180	ND	0.04	similar to mitochondrial magnesium transporter Mrs2p
52	CMM169C	MDAQKVTASAPYASRLDPRTAGVSFP EQGDVQSPAENEGSAVCPPRQTSLGT KSLGANILFGGLAGVTGTTIIFPLYTLKT HLMTDTGSRECRGSSESAAVYNTASK AEPVRGNAVSTGTANPTSSTGPALAT EAAKHTSTLAVKDTACTDSHAKLPTVE AAAPARSSKVASNGSRGPAAASQSTL ASTPRTNALKAASLLKRRPRLV	208	ND	0.00	probable calcium-binding mitochondrial carrier protein Aralar
53	CMM222C	MLVMLGSSFLHPGQEPFHGSIADSSH WISSGSGYRWELGPALSLNVMIASPTH THSDSGVRQTTSAVPERARLVTLSKG SSSTGWCVSRRNITCEHVAFVAVSKP MNEHDETQRTEVDIHTTQAAVLSS	129	ND	0.00	similar to mitochondrial carrier protein
54	CMM267C	MDSCGAASVRLLR	13	25	0.79	NADH dehydrogenase I (Complex I) alpha subcomplex 9, mitochondrial precursor
55	CMM287C	MRRGSLSTTRRNAPHRVSAAELEQLQ RPRSATPPLTAAPESVSHQARRIASGQ RTLPVLPAVGTFSGGSTTRDGPDTLAA GDWTPKLTSNTPLAGKRPERPGTLGK VLTSAAAFASNRLVNKIADAKFGPDLR LDEQTLRRNGLTFAAAVAHEYLEMVTS RRTSITRNVKEKMWRRDSLIQFLEFDT FGRSVLRAMSKEDLIDELRRPVYPAAE ASSPRRRSKVLVEQETPLADALDLDIH PVNTGLTHRERDGIISKCEPVQL	264	ND	0.23	similar to mitochondrial magnesium transporter Mrs2p
56	CMM288C	MSVAGTPQFSELSEENQNSGDGLAKK SPNGLEMTKTSFASEKDTGASWMSST GSRATTHLECFEFFVDGNRKLRSISRL ELLRRAREASADQQVVADVTGLSGMS EAAVLAAVGIDAQRPGEEPLGKKERKR IRRYLKGF	140	ND	0.00	similar to mitochondrial magnesium transporter Mrs2p
57	CMM299C	MLSNSSFCQRASVTRLVQSVQLLAPN ARTWLGRASYDQPLYGAASGGALVR GQRVPAVSSSAQRHRYSTVAGGSLG	76	67	0.81	dihydrolipoamide dehydrogenase
58	CMM334C	MVDEKGDLCLWEHL	14	ND	0.00	similar to mitochondrial carrier protein
59	CMN072C	MVNIVCRRGLLGQLARSGAALLHTVDV GAGSSRRVAVRTVVDWSPAVQQARL PLEANAQSSVQVADERHLRLFDTCRR TCERWSSSQSGTSVTGNDLAGVSLRV FPRRHRAASTATRKCAVVDAAGLRALL QAKPKPPLLTLDWTERVARGRSTSTLA ERWRCSWRARPRIEKAPQRCQSTSP SPSSSSSSSSSSSSSSSSSSSAAGTT GGSSRLFTNRLRGRSQYLGERLHRAL HRVRSWLAQLPARTLKTIESFAKRLWR EVVACIRQPSRLVMHY	279	ND	0.16	similar to mitochondrial distribution and morphology mutant Mdm38
60	CMN127C	MNKWLRPALRTTLRLWRRQWSAEAT SGASVSTGASSPSSEEAG	43	20	0.93	mitochondrial F-type ATPase F1 subunit delta, precursor
61	CMN179C	MLVDRYAGLRALRPIGSALLRPGRVLR CLTSGNGGRAAGEAGSKPTSGPIHPE EGAAAASDGAAESKRPQHSRWRALF RPGRLLGFVALGSAATVLTFWVVDPE GTVARLER	112	29	0.59	mitochondrial presequence translocase subunit Tim50
62	CMN181C	MPLSEGGLYSGFRAVRGSFGGCLQW VRKFATEVPLSPTMRRALALAERTDGA LCGPDASKLVVDRAPVVDSSGHSVRA REVLGVHSFYARGSNFQIRQPAGSQE	104	ND	0.22	branched-chain-amino-acid transaminase, mitochondrial precursor
63	CMN203C	MQTTAALLRAFRRSVQTTHILSRSGFL GCVGLCAVEAPRAANEPVRCGPERRL LYEAAATARLLSSSVTCAGLRLSSPET SSSHLSSVQNGALLAAEALRKSQRAAL TSS	110	107	0.91	cytochrome c reductase (Complex III) Rieske iron-sulfur subunit

Journal of Cell Science • Supplementary information

64	CMN223C	MPQ	3	ND	0.14	mitochondrial F-type ATPase F1 subunit epsilon, precursor
65	CMN320C	MWRTVLVRGSTGALVWRAVTPSLPGL VPSTPHLTTPLPLLGRSFATDALALKD GLIRYRMHYPDGQVPPKVDRNLVRSK WHSD	83	44	1.00	NADH dehydrogenase I (Complex I) iron-sulfur protein 13kDa subunit
66	CMN325C	MRGRLASLVHTVGRTTWSAHLQRFGL AESADCVHRCGVAGHLSSVRALHVQS FMSGRLASLVHAIGRTPRNVHRQRLR FAEYGPRNARLEDSAERQLQASAALR LRACASAPLHSELSRALQQDYRTQVA SGWTRFEQGLKGRFNASRAHEARVR RQNLSTSRSTSEATPTGRAAEGPQHS TNASTNGASELEFRFKLPDAVSIRRLL RLAQPH	214	ND	0.43	ATP-binding cassette, sub-family B (TAP), member 10, mitochondrial precursor
67	CMO080C	MAYVLHRASGLWKHILQRQNPLSGQR RLFEDTRGALGR	38	ND	0.39	NADH dehydrogenase I (Complex I) iron-sulfur protein 18kDa subunit
68	CMO111C	MVIKL	5	ND	0.02	outer mitochondrial membrane protein porin
69	CMO244C	MLRSVSYGTLRQTVRRFRGNFLRRCS SAPESKPGRPSEPTHTTTGSSWR	49	25	0.96	inner mitochondrial membrane protein Sco1p
70	CMO303C	MDENSADARTHARPRTKPVHRSFVGP TSQPETREVQRAVTLL	42	ND	0.00	similar to mitochondrial carrier protein
71	CMO311C	MQSRPDLFGFPYTRALDRDSSSVVVV VVACKKTGYPFCRAISRHGECPELAFR HMGFCVSGYRETRRNLLSVLVQASRR GMCCFEHSHAQLSVGAQRRCRGRWR CAAQRSVHPPCCSTLRRRGASIVLVST AAERVIMSSIWSRLTDKRRSNKPDDAS GEKSDILGAGTSLDSRADPAPDHLWA QVDQVDSVPDIEEENGPRVDFSNLDRI QAETVAPALGIYATPRGKSGEVDYLFA EEYHEYRH	246	ND	0.00	similar to mitochondrial presequence translocase subunit Tim23
72	CMP013C	MPDLSSLQVVGTKAAEESRLESRKPS	26	ND	0.00	mitochondrial DNA replication protein YHM2
73	CMP084C	MSIGSASSFLVQRISLTMRQQLASFLG LLLLWRGRSFLVRRAVKRSPHLHQPA VVLFVLCNVILGWSLLASLGVDWAR	78	ND	0.01	mitochondrial phosphate translocator MIR1
74	CMP152C	MQVLTRLLNGAAFAAGVS	18	32	0.25	cytochrome c reductase (Complex III) cytochrome c1 subunit
75	CMQ191C	MHCARFVLALGRRDLRRAEKSWPSDV WKRAVRALST	36	34	0.86	citrate synthase, mitochondrial precursor
76	CMQ197C	MAMGTTASTPATLETDKKQLLERFAEE EAAFSEIRLAQQTLERCQLLLEQAKAF YERALKSTQRDAQSDTALASTFDSGT VEVQPHSLLIRARLQLY	97	ND	0.00	similar to mitochondrial division protein Fis1p
77	CMQ200C	MVQLWRALLTHRGMVGRFWLHTLTR STVLPVRHGTLGF	38	41	0.99	NADH dehydrogenase I (Complex I) iron-sulfur protein 30kDa subunit (NADH-coenzyme Q reductase)
78	CMQ258C	MEAKAGVATADAAMPALVLTRSTHSG FVLLSSCVENAKRLYFWGVLCGDMVV SGHNEDAKLID	63	ND	0.00	similar to mitochondrial carrier protein
79	CMQ270C	MQSIACSGTVQSLRRLVSVGTLGCAQ ASSRRWLA	34	33	0.95	mitochondrial chaperonin hsp60, precursor
80	CMQ323C	MSATSHACPPA	11	ND	0.00	V-type ATPase V0 subunit c
81	CMQ441C	MEAPSNVVRVGPDRQSAQVALSETLN AEPREACLGFLAQTWRYRTATASRST WFVSSRFRNALPQPSRTAPNRGLKRR TVPVARASPSERSPAHADSGGTAYPR VGAPQPANR	113	ND	0.00	probable lipoic acid synthase, mitochondrial precursor
82	CMR073C	MRRRCLASCCRRRYSITVVMNETRTN DPKKHRTTERSLPPSESFRVGL	48	14	0.94	probable mitochondrial carrier proteins; Pet8p
83	CMR158C	MWGSLRLLSRIRVRNGLPEASTGRPLI NLRCRSMHSSQRDNPATQTDVAGTQ RALSSVAAAVAATAGSANANIP	74	33	0.70	mitochondrial Mn superoxide dismutase
84	CMR162C	MSASGEPVTLPDSSAARMSPPSTAHE PAITDLCSWS	36	ND	0.00	similar to mitochondrial carrier protein
85	CMR188C	MAPFPRVGALGIVRQWFSHVRRGSG GYFEWNPRRALAGRSNV	42	36	0.63	NADH dehydrogenase I (Complex I) flavoprotein 24kDa subunit
86	CMR213C	MQASTAG	7	ND	0.00	mitochondrial intermembrane space complex subunit Tim9
87	CMR248C	MLGCLRGFASKHATRSGRVLRLDTLG NAGARHTGGASALEERTSASCAPSRG LYTACFPGAVAMTTATCVRWANDHRR LIHSKRWPRNPVRRSVSNRFDRAAAA LDLLPAAEVGSAAEKLLERVLSEVPTA VAATQVAVAAQSTPQVAVEVPAAQRK PTPVATGTRNDPATALLSSALTPGPPK GYVYRTRPVPVPFSERRSETLARLPT TLEAAERQAARELERLWRNAPPELIAS DRQWKLLLPPHNSFALTPASNARLPN VS	266	ND	0.49	probable PET127 (probable mitochondrial translation system component)
88	CMR289C	MYSLTKLSRQLWKLERTSVRSFFVER AFTLGTRSTRGRLSTSASVSSSAQPAT AAATAPEPPKPPPKPDH	70	39	0.86	NADH dehydrogenase I (Complex I) iron-sulfur protein 75kDa subunit
89	CMR309C	MASWRSGAGTTLPVSWPRNASGSSP SLSTEVETGTPAAASKLDRHQTVHAER SPTISLRESAFSVEPVSSAAVAQSTCP RKPQTFAEPPAPSPKNAVGSETSVPH	141	ND	0.02	probable mitochondrial iron transporter Mrs3

Journal of Cell Science • Supplementary information

		EQIERRAPVVHACAGDALGRTTSTRA RLQDNLPLAL				
90	CMR404C	MESLSTAPRDE	11	ND	0.00	cytochrome c oxidase (Complex IV) subunit VIb
91	CMS091C	MSSAPAERLEPWKDL	15	ND	0.00	probable mitochondrial carrier protein
92	CMS209C	MSAGTSPVSRKSEEADVVKPRLPPNL QF	28	ND	0.00	mitochondrial inner membrane insertion complex subunit Tim22
93	CMS223C	MNRFTRSLLQSVYAIPQTGLSRCCFGS WSSRAVLDAGQRVSASLLGTRQELRL TQQREQRVLRHSFSTQLNPGGTERAQ RS	81	66	0.78	NADH dehydrogenase I (Complex I) iron-sulfur protein 23kDa subunit (TYKY)
94	CMS272C	MRTLQRTLTCLRACAQRAFVARWRHG LEGSPIRAATSEGHAGLARRHHWRTL STANTMRT	60	52	0.97	isocitrate dehydrogenase (NAD+) subunit 2, mitochondrial precursor
95	CMS327C	MVATQFITRFGRANTLIHRSPLGQTVR RWASTGGDV	36	29	1.00	pyruvate dehydrogenase E1 beta subunit, mitochondrial precursor
96	CMS342C	MVATDKAR	8		0.00	V-type ATPase V1 subunit A
97	CMS359C	MLFESEKVASPGSGHGNGSWAFDADV APGPWLHTLIGQQPLRRRSSTSSTRW CAHTPIWSLERALVMPSSPPDRRKRP ADSGASPSAHTRTSSHAPATPAQKTP LGLGRRLLMEASAGAIAEALVEFLLYPL DTLKQTQQLPTAHRRLIHSRHATAAAA STLAWRSASGLHWLGPVRAFQHAVR RMADGRGF	191	ND	0.00	similar to putative mitochondrial carrier protein
98	CMS372C	MTLVTVARSLARALGFARQGSIGGWY RGALTSTRSWSSEAGSG	43	36	1.00	mitochondrial acyl carrier protein precursor, NADH-ubiquinone oxidoreductase (Complex I) 9.6 kD subunit
99	CMS431C	MNDDHADLKTSSAPYRAEASEAGNHD GNPK	30	ND	0.00	cytochrome c oxidase (Complex IV) assembly protein
100	CMS453C	MGPLTRTPLLPTLRRPEHCETTARSYP CRTRMKLYSERGRNPDSTFGFTSDRC SFRKIMARTSEYDDFMNEDDVIAVCAA LITATSPGTGGVYHGAPEAKLSGLVAF ERLGSTLWTNRVSLA	122	ND	0.01	similar to mitochondrial carrier protein
101	CMS471C	MASTGSAPIEH	11	ND	0.00	mitochondrial presequence translocase subunit Tim17
102	CMS501C	MEKRETQLPLW	11	ND	0.01	similar to mitochondrial carrier precursor
103	CMT198C	MRIFRAFPPWKTLKRSFAPLYWSELAS ARIPVLASETGAVASALLFRRTLCNDT GNGQTRDRARVYLSSGEIDWSQPHPV GYPAGLRPDPDATAKLRAAYRTILERV QTLAPESAYRRNIELLTRYRLEVTEAHL DDRKALEDKIGAGLVEELLA	155	51	0.61	NADH dehydrogenase I (Complex I) alpha subcomplex 5 (B13)
104	CMT209C	MRRLLRGPLVAELASQVSKLGSRNQH TCKQFQQV	34	51	0.90	succinate--CoA ligase (ADP- or GDP-forming), mitochondrial precursor
105	CMT234C	MRSQLLLLRNVLRPCWRPGAQQ	22	26	0.99	cysteine desulfurase IscS, mitochondrial precursor
106	CMT256C	MHLIWMALRASSRRLWGVCSRGRLRF TCAAGSTDSASAAVQRERVEAR	48	26	0.97	pyruvate dehydrogenase E1 alpha subunit, mitochondrial precursor
107	CMT361C	MLVRFSQLVTWSQTPMVTALQQRKDN GGSNCGVDQVLRAAPSTLPTVWGALG GRPATLVACSADENPTFGT	71	61	0.18	mitochondrial phosphate carrier protein
108	CMT412C	MVHTLLRQAATCFRKCTLDTLCRGQFL RRFVSGSAQGHDITDVRRFVSPALRQ GSPIISPTSIDGDVYNVYPAATAV	77	30	0.98	isocitrate dehydrogenase (NAD+) subunit 1, mitochondrial
109	CMT434C	MNRALRPFARALVRSSDLLVRGRAAV HGALWVTANRDQRQRSLSSWRATGA VRRSALSA	59	55	0.92	mitochondrial F-type ATPase F1 subunit alpha, precursor
110	CMT452C	MTYWKDGARLVQWKWVSLSRQLFPS LGPVRSFLAHSFSLKRAGSTPVRLKRT LRSDKANQGSPGPTTDNAARREASND PTPADAGAPPADLFPPEIYGEPLNYTN SDHDALRRGVSNAWSLRDLRAAALWP AVARWPQRRTRPPIRETPTGTPFVSQ FFELPPGHMVRYLEDKGLEVRMMDQH AVVRICPFCPPVRGKADNMFKLYVHTS SGVFFCHRCGAKGNHWDLKRSFGDIL EPLSWRGQFPENAHLAPGAHPTSARP ADSGAASVSANTSQIDSVATEQLPVVD AALAAQCEARLESSDAVKQFLKSRGL QLATARKFRVGAGRFRFRDSGSTADI WSEHDCITFPWIEPEWLTTRAPSTLVE LDHRRDPNPAGSNLGSEESACQDSSV PPPSRVLRLKIRSVLRKSAMRLEPRGG	421	ND	0.22	mitochondrial DNA helicase Twinkle
111	CMT541C	MLIFLTPVECIYERPAPTYRNKAFTTPE VRVPHRERLHRVRRRALTPVRVPGVV AIASDANEGSTPSDEPWWRERARLVV SRQSQATGDAPTSRRRFLQALVLALV GFVAGSLGKSASSGKAGTQAQAVTLA PTPINPYARRRQKKQYEKYLEIQKALIE RNRLRRKYSRKPPIDTDALPYSLSALS AFV	190	ND	0.00	similar to mitochondrial carrier protein precursor
112	CMT561C	MPLAALGQRAALFATSATTTRSLLGRL RESGPQDHRFVSTSTRLVHQSSQTFG ARRATLQQLALFKAVGTGGSYGVPGG KQTLK	84	88	0.43	aconitate hydratase
113	CMT582C	MTLVHSLRVFLSRRSFTKEAARRLPRH VTSIGAFPREERLLARSKQRIIALVCGQ QRRALGQLVRSARPAV	71	27	0.92	succinate dehydrogenase (Complex II) flavoprotein subunit precursor

ND: Not detected.

Journal of Cell Science • Supplementary information

Table S3. List of 97 putative chloroplast TP sequences.

#	Gene ID	Estimated cTP sequence	Length	Length (TargetP2.0)	Score (TargetP2.0)	Annotation
1	CMA030C	MLPAFTFSVTAPDSRSFSLHHRWSARGAVRFVTPHRRCRLWRGRRLAAGDLQMTLAEEAKSPSNAPRSHQKLVSELFPERFRPFLKALDDGSLPLEDIPAFNAR	104	46	0.76	pyruvate kinase, chloroplast precursor
2	CMA048C	MPSPALTLSFVCASAWLSDRHNISTFCCGQTGRASTKPARGPRGLGYPRSVGNAQPLRFINANLPDPRTKPLASHHESVRSEDE	84	ND	0.38	3-phosphoshikimate 1-carboxyvinyltransferase, chloroplast precursor
3	CMB021C	MVPMQCMFVANGFVGRFDASGRLQMGQKLSRRTTPGRSSVLSVSMA	46	45	0.64	chloroplast chaperonin CPN60, precursor
4	CMB025C	MLRWSGSQQVTDRNRAEQHGLVVAFAFPARAPRHSEATHERWTGLAQRERGLCVPLKATKWKRDLWHSRKLQMQEPAQRNR	81	ND	0.01	protoporphyrinogen IX oxidase (PPO)
5	CMB032C	MFVTLTASKIAAVPYHSFVAGRGVGTCAPTCQRPASDTSIEMRVR	45	31	0.61	chloroplast ribosomal protein S21 precursor
6	CMB093C	MKGGQSLGAAATKDIDLCFRTNLEKNTAQSVFGANPWPAGHISRQKLLVSCSYVEHCAGQRVSAVTEQPLTSACTCRHARLLFQWGAPRRVHASVHAYRYQWRAQRRSRASFWVQPRSRSRQRRLLVAVQQTDASQQQSANLAKVASERELVGATTTPSPVVQT	164	ND	0.00	magnesium chelatase subunit H
7	CMC133C	MFVTSFQLQPKGKGSFLARSAGSRSSLSHCGNSGGTVAKLENAALDRRQLLQGLLVAAVAVFAGKAQPAHSGSEPKMSFFGADAPSSPFSYNESQKEPLFKGITPELLEEYR	112	30	0.11	similar to oxygen-evolving complex component psbQ
8	CMD084C	MGTQSLQAFVSSFAGVQLNVVQSTPAFWGGRVREVSQGHRPASPCWRAGRFSLVAAGDRKKKNPGSKRTPPKSMRREKDTQNGRTDTDPEK	91	55	0.95	chloroplast translation initiation factor IF-1
9	CMD190C	MQRSPVARRHRMLHLRMGPKQSQIA	25	ND	0.01	NADPH:protochlorophyllide oxidoreductase
10	CME041C	MFTAAFSFSLPRSAYGKLSKSGLRGAGIHAKGSRTSRQVLCAGTGDEPRFAGLDEFRKDIEALGLITEPADTDFARKFAPFRGKPVR	87	41	0.97	photosystem II biogenesis protein Psb29
11	CME148C	MLARERRCCAGMFAPPCPSDRVLVSSNCGLNCAQLHQRFGKQRSALNSRRWSRPRGRWGGIALGKPRYPKALAASWGASGLCACSQPIHQSVTAIGDLAHEVSSLRQSV	109	ND	0.10	chloroplast peptide chain release factor RF-2
12	CME194C	MSRLAARRSPDARSRCRSPTSRRGRRRGCGTAQRQTAEKAEAHTRPMFLLPSGTLETVFCGRRAYTSRPKRFFSLKAVEASGQETASSSSAAGERAQAMQ	100	ND	0.02	uroporphyrinogen decarboxylase
13	CMF117C	MVLGFNVAGAVRTTAVRERPPVCGVGAQSSRFRGDTMVSVARTRAFLAVPPLRSTRLPKPTRQTWLHMQTSTMQKARYAEKAPRGVHKEAAVGAVKSEALQRKNIQ	106	78	0.54	phosphoribulokinase
14	CMG019C	MHTVPEGCSSPRADEQTKLMRQQPHENGTKPAILSEASALLAAMRHWEEQLAQRQLPID	59	ND	0.00	nitrate reductase
15	CMG110C	MRLRGRLLLAKADHPAASLELCVRMAFLASSCVTTAHPLTRRSAKQRCAAGVHVRRALLASGAAGERRLRRVQH	74	59	0.00	probable phycocyanobilin:ferredoxin oxidoreductase
16	CMG111C	MDLKEHTFGFANSPQGMQWATTSLRQLVPRNLVSWSTPWSLRDYATGTLPRWKWCPRINRYAKRERALSRPQRSARPALIATLSPEATARKKAAERVAVGKRLS	104	ND	0.08	alanine--tRNA ligase (chloroplast)
17	CMG162C	MFVTTPLSLKNSKVSTRSSCCVQRARRSTGVAKLAMKEAW	40	34	0.65	Rieske iron-sulfur cluster containing protein
18	CMG217C	MQALFVSVPLYQERHIVCARAATRAPFPLRTPRNGCFAAGHRHHCQHPLPLVVQRLPVARRHHALHLRM	69	93	0.52	NADPH:protochlorophyllide oxidoreductase
19	CMH024C	MKRPVSRSCAFATPLRVVCVRHGLSLTHREGSYGCRGPARFSVRVATSMSLDPGI	55	ND	0.19	chloroplast peptide chain release factor RF-1
20	CMH047C	MFCISEPFTLCASHTEHHRRALCGKQSVWGCFTNTTSWFGRNRGVRNSAFLAGAADAFNSRSKARNLSRRASLSMKLPMSCRADTIGSKRCLVIGGTRF	99	81	0.53	similar to mRNA binding protein CSP41 precursor
21	CMI040C	MMLGFTTTFRAFQSLPRGTYRNRGFLGVQRGSCTRNRSKTRVGHVLVAVSEGVGAGTGTGAENAASTSTQIVVVPRTGIVQNREAKVEKSIQQPQLATAAAAAAPASRLWGTPLGALVGLALGGVAALLASWQKGSQKKDGLQKPEDQAKEGLVLKADASAAGSSAYGTNGDGMVGTNGSSSPAPPSREL	190	48	0.84	chloroplast ADP,ATP carrier protein
22	CMI049C	MEAFVQIPVNSSFFQQNVKLNLRQKSLGAPRARVARSVTLRMR	43	42	0.78	fructose-1,6-biphosphate aldolase, chloroplast precursor
23	CMI112C	MFVSLTLTQSLQRAGIRAGARQAQAYPSQGFEQAVPARVRYGRQAALLTVKRSHGAAARRSLFTLRSAGVTE	72	67	0.82	peroxiredoxin Q
24	CMI113C	MSSEQAPTKG	10	ND	0.00	similar to peroxiredoxin Q

Journal of Cell Science • Supplementary information

25	CMI196C	MKAANLAFASSFLTHSSRFVFSARKCI TEQRRLSQPQTGKRACRLVRMSVV	51	48	0.99	sedoheptulose-1,7-bisphosphatase
26	CMI248C	MAFISTPFAKVSAKRASVSANRRALCM RSDADPVVSRRALLSGALAVAVAA	51	54	0.19	photosystem II 12 kD extrinsic protein
27	CMI251C	MFVWSVTSQQLHTLRPLGAASSSWLE AGRSLHSGRPRALVQRSWRVAKPPK RVQVARWPRMVAGGSGGGPDGNLL	75	61	0.83	heat shock protein ClpB
28	CMI281C	MFVLTTNSITFRTVSRCHPGAVERQPT TLGRKRVALRMTFARPSTVDDARGGS GSGGGSSGSDDDY	66	40	0.57	cytochrome b6/f complex iron-sulfur subunit precursor
29	CMI290C	MLGFVSGTASLTKKRGGRSAQACGSA VTRLRLQAGEPQPVLAKGRLPSVTAFA AAVLLAAATHSAVLEPVQ	71	34	0.16	manganese-stabilizing protein precursor
30	CMJ042C	MVFTCAAFVAPVGGFRGTAVRATSRE AVGPRLQAGDQPGAFTARTRSLGVPL TRSRQR	58	85	0.99	glyceraldehyde-3-phosphate dehydrogenase
31	CMJ044C	MFTTWTAHCVQRWRRSAAAARAPRC LSAPTARTWAVASRRYRPAQRLQGPA RTEPRMSGVNGGLAYDIDEQQLPDQI KPLVQMIVRGPDSPAAFIDAMERIADW LWQQQQSANDRGTRPPDTL	123	34	0.29	phycocyanobilin lyase beta subunit
32	CMJ054C	MEGLTAFVACAGVRRAVLSCSARFCS QSKVPNAYNAGTKLLVTACRGGARLL VADAASEVAAKAEEPPARGSLESRQK QNGSRTADDR	88	24	0.38	glutamyl-tRNA reductase
33	CMJ151C	MTKFAPALDDLGVVAPAPRQRYVTVA FIGTGVQLGTRSAISNTGRFLWERAFA GSDAKRLRVRHQCREGRLRLGSPVAP RASWGTPGQRLSNLTQSLAVGLYMVP LLFCFVDPMGLFAAPSPAFKLPPVDMT DPNRCNLTSSTIGQANAARDKFLDGRF CD	161	ND	0.02	similar to thylakoid lumenal 17.4 kD protein, chloroplast precursor
34	CMJ154C	MATSKPSVTFVSAPAQFWGASSARPL APRRVHRPCIRRVPFLQAK	45	44	0.99	geranylgeranyl hydrogenase
35	CMJ188C	MVLSNTAFLALPSTPVRHRGHRSCCS RRPQAVNWLQRTRACAASSGRNPAF SAGTSQTAVLPLTRA	66	66	0.99	unknown hydrolase, cbbY homolog
36	CMJ305C	MIAFVINSATKAAVSPLKGQICRGLPKN PSFAASTWLGQAALSTRPRSSQGRGR SGVRESAATRARAPVCVQTSDVAMRV	80	70	0.98	phosphoglycerate kinase
37	CMK044C	MEGYVPVPISNSRAGRWTGTGSDGTR VFTQARDVCTRVPVLDTQLCMSHGAH PISKANVRLAFQKPGGVLGQVNVSVFA QNGCEIKPGCGNRVAVSKSEAHALGS ESSCFTHGSRCLYPHPQRKSAQENVN SVDTDRGDTRMIAVGPRRTNARRSDN PEKLEDSCESDSVITGIILRSPDEAYQD RGDHAGLEHAVASRNQRRNKIPRVDP RGLAAYADELLAAADIDIISLSAEFGSV DRNSRNQRTRISFPGSLRAYVRGLER QYQFEQSSDAPESVAEAENKMVKRSS PAAKGKSSLTRSEKRAAAAKTKGRQP RARKTERLTGAQVRMRAGPRMKNTV STGDASVASVNSNSGTVDPFDGIFSGL DDEEPDLDADSDKLLDA	386	ND	0.00	sigma subunit for chloroplast RNA polymerase
38	CMK050C	MFVANLPETVYSRRGVLKSPQKSGVC WKLPKTANAAPVKYLERDRRFRKPQV AVCLEAGQRLSQQ	65	ND	0.18	lycopene beta cyclase, chloroplast precursor
39	CMK058C	MTPPFFCHTFCSAGAFVPVNVSHFRA QLRKQETCEELASRDAWGRSRSRGN CLHGKNLPSRHRQALLLLGTATAGSSA EVRHGSPKSRT	89	ND	0.25	geranylgeranyl diphosphate synthase, chloroplast precursor
40	CMK061C	MFISLAGDPCFVRGKALKRATGPVSR GRVTARKSTGFLALALKAVDGRSPELT PSSDELATPANRNGISEDAAAKLHS	78	41	0.83	DNA repair and recombination protein RecA, chloroplast precursor
41	CMK151C	MSVAAAFEQRLESGVRVNSFESRPLR YSTSRGLASEQDSAFILPCGVFNKHLG AWCPGRVGSTRLHAYRCGSVRPRRL CLRGLLGRPVPPLQAPQPVGIPGKE	103	ND	0.12	phytoene desaturase
42	CMK176C	MIKTRVTAFVQLPVSAQSGRAARVNAP RKHRLQSRKFVLHSELRVDRRVFLRN SLAVFLGSLTAAFSASRTDWGAQPAN AEVLRHAAPGYE	91	41	0.20	photosystem II protein Psb27
43	CMK187C	MMRHVPCFLYSFLGNSSTARRTDVQR ARHLQAQLSTACWRRAQRTRTAQGL GDGCRVAFWRGVPRRPVAERWWSP RMDV	79	ND	0.19	chloroplast translation elongation factor P (EF-P)
44	CMK291C	MNTSIYESRIRGEKSNDTVKQGTPEAK RCFSVGCSGRTGGVADACPHASTAC GTMCASDQARSDAQACDKVLTDVSVL GGTGHQAMITNRE	91	ND	0.00	delta12 fatty acid desaturase, chloroplast or ER
45	CMK307C	MITALRRYAFEHRNEESVFLLGCRAM WSQLAPVHSLQRLERKFRARCRSRKP LLRLHHGSGDGEFSVSRRELLKHSAA ALTAAALSQLGVAALTERHVVDEAAAA NRTLSGASGVVNK	118	ND	0.03	probable thylakoid lumen rotamase
46	CML047C	MAFAISIGFSGTKMQRSSVANSRASSL RTRRLHAFAGFPLVGARASEFSGAHP LGRRQALRSMGQRHGIQGLQCRS	76	75	0.88	similar to mRNA-binding protein
47	CML164C	MYIVESSSVISARNWNQTSSTGVSYC QSCQALDAVENRRAKLRASFTLLKTRF CASARIRGYVAGSIYSFSKLHVRCSSV DSGEHGVSQERVHARPNAAFLADVDS	150	ND	0.41	DNA mismatch repair protein MutS, chloroplast precursor

Journal of Cell Science • Supplementary information

		VPAAATTAGAAMGVTGENLGQTVVSR ESNTASLGMEQVEQVSLV				
48	CMM019C	MWAFLASLPLRSRSDFVSRSVRLGTP RSLLSARTTRPLSASPAASLVAAEERA AAPGNDAIKVITDASTSSDTKTRAPSRF ERMRAKYGLSDVSEPEAAPAYKELDQ AVQNLS	113	49	0.56	chloroplast ribosomal protein S1 precursor
49	CMM072C	MIQRSGSGRTSMRVGFTVALFTGRTR SWFRRRGIQPSQLVDLSVQRRVPAVR SIALDGIPTSKPRMQSPSEAERPSART EGPSGRRSKRARAPRSRSKSEKDSSA SKSVKTYAEPDQPRKRSLEQASAEAS LPARKETEDNSAKKGGRKPRKEKLEA LTELAKERLREEQRKRLEVQQMEREQ RERAAAAQREGQSDDVLRERGRSSK YDGPVETDDDNDEDDDDEAMQLGER SLTLDAMAEPSLATDDSVNTSSSSSSS AGERQ	265	ND	0.00	sigma subunit for chloroplast RNA polymerase
50	CMM158C	MRILSCSLLRNGYSGIRSSMNSGQIAF SNAVSFPSLRSIQVRRISSVSVRSANK VRFSSYFVQREAYRGAAVLRLNTQAP TRRLVTMAIDTQT	93	44	0.03	chloroplast ascorbate hydrogen peroxidase, precursor
51	CMM166C	MWQLCLDPHISRSSSPVEEGCCTQRV EKATRRRRALQRARHTMYCRRVWTA HLEARSARDSDWKPSSGQTSDPRQLK GEWRSSAGRDASKAENAGQSQSDDC NSVANGASRGAPKTWSGEALHPYGIL ERAVRSFRTSEPAGSRAQDTVDEVFA KQPGAWETPPLSSPTNEGLDAADALL ANDASMGGSFLTLPQTTPKRVFPWDT TTGVIGLSHFERQVAFYRRRQR	228	ND	0.00	phytoene synthase
52	CMM178C	MFLSFSAVFAGRNCCRTQQSHRQSVF SPTAWPHLFSGAQRVERGRRATSQG RRARSLLSTRTFCMSSSQEDQPKSKS MASLRPK	84	63	0.36	NADH dehydrogenase type II, chloroplast precursor
53	CMM190C	MPSLRTLSSAWRYHKTLLGVSCWTPR STLAKDVSTRFTGYLSFSQFVACVRVC SDSRQMLSRITLGFVQAPLARVAPASC VAACRVLPARPASVFRGAPLIPSVPVP RFNFAVQTRVPAGSGVLSLEAM	129	ND	0.02	chloroplast ribosomal protein L17 precursor
54	CMN203C	MQTTAALLRAFRRSVQTTHILSRSGFL GCVGLCAVEAPRAANEPVRCGPERRL LYEAAATARLLSSSVTCAGLRLSSPET SSSHLSSVQNGALLAAEALRKSQRAAL TSSS	111	107	0.02	cytochrome c reductase (Complex III) Rieske iron-sulfur subunit
55	CMN234C	MAFISALSSIGLKTGTVTRVACVTRVPA RGLRMQAPSGATMP	42	33	0.91	similar to chlorophyll a/b-binding protein, CP24
56	CMN252C	MFILSAQRLSGTQVFRRLAEGCTGYSV SSGCRQRRRCCPGAPQRPRRVRVNA LQLQSGLADPLDPK	66	55	0.06	similar to CpcE-related protein
57	CMN290C	MFLAQTSPAPSRVAALRPSTSSERKQ HSWVAAGAPSQRLFSSCSGERTERPA SALPSSGATTGALHWTRRSLIQTTLLL AAGCVAGSPALAEKATAEAPAVPRDV RNQLEL	111	43	0.10	photosystem II oxygen-evolving complex 23K protein
58	CMN338C	MQALFVSVPLYQERYIVRARAATRAPF PLRTPRNGCFAAGHCHHCQHPLPLVV QRLPVARRHHALHLRM	69	ND	0.37	NADPH:protochlorophyllide oxidoreductase, chloroplast precursor
59	CMO066C	MFVLTTNSLTFRTVPRCYPGTVERQHT TLGRKRVALRMTYALPSTVDNARGGS GSGGDGGGSSGSDD	67	40	0.43	cytochrome b6/f complex iron-sulfur subunit precursor
60	CMO089C	MPDFAFVTTVHHKFNTHSHTVCRNKS KSRCRKGLANVESLLDAHGDGLEQRP TPQSVLAHKALRAGQVGLSSSSRSDY QQKPVDTDTPPCVVKVFGVGGGGCN AISRMLEDGEFRGVRFAIANTDHQALIE FKKKYILYTQNAVLETVVPLGESICRGL GAGGNPEV	167	ND	0.00	plastid division protein FtsZ2-2
61	CMO121C	MEAYLSAPSRPSVTLSEFSGRRVCQT KRNDSLLGARGRFAPGTWERVVLGRR GGSRTVSRLPQVRATATAGVSLVKPK QTGNAFA	85	66	0.88	transketolase
62	CMO128C	MLAYLSAPSRPSVTLSEFSGRRVCQT KRNDSLLGARGRFAPGTWERVVLGRR GGSRTVSRLPQVRATATAGVSLVKPK QTGNAFA	85	66	0.99	transketolase
63	CMO245C	MAKQAPHAFVSLGAPKLRSTNIWGRV SVSVLPLETRHQRRFGAVRLRAALDLP M	54	48	0.52	fructose-1,6-bisphosphatase precursor
64	CMO250C	MFVQTSFFGTGVKASAKSAESQRCLA HSSWSVRMTGYDMNGSSAGNLGPRR IKSSGVANDR	61	34	0.51	phycocyanin-associated rod linker protein, fragment
65	CMO314C	MTSVSVAFTNVAGLLAHGSKSRCRNA EVLRSRSAAERASGDRQRWRICMSD GERVHAYTETVVESQLGFSVSRRALL HHLALGALAVALPRSGALVAAPLAETV RGAA	108	49	0.25	photosystem II stability/assembly factor HCF136
66	CMP052C	MRRPPATFVVPSRAIRNTGENVEYRN SLSVENLGKHLWKERRQRRRYVPPLP DANRHSRQRVPVAPARCRGLLLCCQD CSEYELKSFVDRDEDEHGTGGGRGES SGSRSSPNFISWRQ	118	ND	0.00	DNA mismatch repair protein MutS, chloroplast precursor

67	CMP166C	MIGFVGSVSSLGARVSSFTASCSASNSCSVVAAPRRAFDLRAAEEKPQTSSSSAAGSSSASSSGAGSSERAAPSAVSSPPAAATSTQSGAAKMPTMTALKTAANRKLGEVTQARLRKVGDSVTPSYRQIDSFRVPAYPIPGKDDEVSRRTERMYM	155	ND	0.38	phycocyanin-associated rod linker protein
68	CMP227C	MAGIGSGFVPTYTLRSARGSFGETKSLSQPARTRGHQRLTFVSNARRRCQLVASAAEERPERSQTQSEIDFAKLQFSEGNEL	82	54	0.81	probable ferritin, chloroplast precursor
69	CMP284C	MDSETPTKNEAEQRASPDRTGQVTQRPVSLFRKNGTFAYPLEDSDVYEEQVL	52	ND	0.00	similar to chloroplast outer membrane protein Toc34
70	CMP285C	MTRSKFTPGPFPCGVMFVASNSIGTSLFVTGKTHVRHLQPLAKNAGSCSAKNTRRKSRMQAGATTLKAVASERRLC	76	ND	0.44	glutamate-1-semialdehyde 2,1-aminomutase
71	CMP289C	MFQSALPTRRQSRHCKNLSRQRCLRTCIASADPESRTRPLR	41	29	0.08	similar to phytoene desaturase precursor
72	CMQ087C	MQAFVGTFTAARGGFVSARRDAFLCGAGQVRPAAPVVQAAPQTLVMSGKTT	51	39	0.61	chloroplast F-type ATPase CF1 subunit gamma, precursor
73	CMQ142C	MYAFVSFAPLVQRANTVSKATGTSAIRSRHASGYATLKMEQ	41	30	0.23	similar to light harvesting protein
74	CMQ165C	MFVLTTNSLTFRTVPRCYPGTVERQHTTLGRKRVALRMTYALPSTVDNARGGSGSGGDGGGSSGSDDDY	69	40	0.51	cytochrome b6/f complex iron-sulfur subunit precursor
75	CMQ167C	MVVVAHLLYVTVSVHATLDEILSGWGRNLKCVLDRRRPGLAGPRDTRPRPRSGGHVLDALFARKLPFPPKDSFDGWCTAPLQTMASLP	88	ND	0.08	sulfate binding protein of chloroplast sulfate ABC transporter (SbpA)
76	CMQ172C	MFVTLGVPSRAVSSKAESYAQRLRIVRTARTRLFMSAQSA	40	ND	0.26	triose-phosphate isomerase
77	CMQ213C	MFLSWARVSSLKRQVAARVCPVSRVARGHAVMTPHMKVTPLGSLPESLRSLSTLVSVPNVPVIKDENKVLAQE	73	51	0.05	sigma subunit for chloroplast RNA polymerase
78	CMQ292C	MAFLTFPRLSCGRGEHLRASLSTQSAWCPVGSTRRAMLHAGASPFLSSWRLASARRVATGNSVHSGLRELQAIRIVDKEGKIPEEIANENFTL	93	54	0.60	chloroplast ribosomal protein L15 precursor
79	CMQ295C	MFCAFAFFPATLSGKHGFCGAGGALQPARSRLVETPFPGTRARQGPSRVVSKDKLQSGYGVWMTSSLGRIGRLAQELMRDNMVNGTADLGRYRQEFLAIASEADLKGSKVSRTLQRFAAATLAAMIVLHGFEGGSGFPLPVQAAPMTTTTTTTTTKVDANRTAPATGERDTAPVPAGSARGAKEMNSRNVHVR	193	51	0.54	cell division protein FtsH
80	CMQ393C	MFHVTYPFTQRQCFLRSREACLATLPAGAFRKHLWRPSCWSFRTRLRKEASLRKSTVLAPLTRRLQLSLFGLPERFVRKSKSPVSAESSVATELTRDRVKDPTL	104	85	0.54	chloroplast preprotein translocase SecA subunit
81	CMQ461C	MSTRRPAADAWSSGGDGMHSVHSINIQIGVCFGAGPFLGQRRLPAAVPRQHRCAAHVADLRRMKSPRESVSFIAPLSVRVAQASSPASGRWERPRLLGKGAPLVPLRRRPQARVHAALRLRCVEGTDSLKGIQVD	135	ND	0.12	chloroplast ribosome releasing factor RRF
82	CMR052C	MFAFLPSITSHRLYVSGKDGLRTSTLKTESARLQTRVGCRSRPATLRMVAA	51	50	0.87	ferredoxin-NADP+ reductase
83	CMR079C	MEQHTARRGSSEHLSRSRGVRATARNATSINGNEGFYAATWNVVVVCKRVPVPCASALSNLCWISQPRLWIQSHSSNRGFGASVCTQPTGENRHVRSSTHPMDYWLRTRTHCRQTRRRSRNRARFRVGAEPLATPTPNRQTTLYGRCGWQWAHGSFQRVRATKFQTLGESTCRLVLATDGDGRPKRSPCHWQRMRMLGEPASKGCTAAETSRMGSLMRHSLTNLRLDVPSLVDTGVRWACALVISAGVTLCGVHPDSVAVGRASAAEGASDSIADFARTVGAAQPRTSIDRFGNPLLRRYVST	303	ND	0.00	similar to thylakoid lumen rotamase
84	CMR165C	MFTQGRTASAEARCCRLSSDGVGFVTATLGLWRVRPLKQLDLPRAGQRLQHGKTCPGRGVRLVVAEPRLLRSCRCCANTNRSRDLGAEYALEKHGFVDGARKLEPEANSVCAPAPLGPVARDEQREHVPKSVDSGTTPDASSGAQRTPERTGSSRRRQARAATLRVGQSTSAVPDSPLQTPLEETFVSVDNRLPVPNWEAKHPGGPGQAHMRETTSPSAKTRSSKSVRPARQRQLSNADQRLSPTSLNDAVHKLPQGSRRSLSTNPSAVVNARWERIQDSERAADDDGPSIRELDYSEDDPSDNEPLAWETYPETPRARSSESST	325	ND	0.01	sigma subunit for chloroplast RNA polymerase
85	CMR278C	MKRTAFAPGLPLNRRRLVRTAPRATGAGTARKPAQRPLVAGTTASNGAAPVRGIATRKRRGGALPGDPAAPK	72	ND	0.29	probable ferredoxin
86	CMR315C	MKAAFLIVPSNATHFSSVSVSKTRATFAVRRTFLRTRKTTRVAALRMANEK	51	47	0.77	CP12 protein, chloroplast precursor
87	CMS004C	MNHRTPLSARGEARAAYAAFVPATGTVWSASRHRSLGRSYFTASRPGTLQPHCSSRGKRHNVDWLRSAMRVQANGASYPGRGNDRQLSSLP	91	ND	0.08	plastid division protein FtsZ2-1

Journal of Cell Science • Supplementary information

88	CMS299C	MEQSSAFIATLGAGRSRGSYALSRHA CATAGNKNLWQRKKSLSILGLKVGRG RLCLRAQTSTGAVSNATEATAERPFK	78	58	0.80	biotin carboxylase, chloroplast precursor
89	CMS393C	MLLFANPAGIATFNVSKPLSNVFDLPRI ARSYIRARPGAGGSLPGSASAIPTLRA RLRRSATGKAACGLVASVADVPKV	79	71	0.97	chloroplast 3-oxoacyl-[acyl-carrier protein] reductase precursor
90	CMT061C	MCLQNGDTWTAFVAAPLGRTLAPAPV LVRCRTGLSTGATSPRYATTGQAHDN NARVYVRGGVRLRTGWRQSHCSMHQ DGTDAFRVIRSARELLEARGYEPSEFG LRPSGEPR	112	30	0.48	zeta-carotene desaturase
91	CMT063C	MGALQILSTQRICGRLLEVSGRKLDTE AEANDTNSIARSEQRLLFRFVPVLATG PTAGSCGCKIGFVAGLYASGTGSVRSL ATASRQCGYTAAGCGLGGAWRRCRL RPGGEPNRRRVGLMSPRRRSVGVQS LRLQFVDPSQSLAALQDSWHTVTFALA SASLLLVDAADGSTSAAAAATAAAGAQ KAGLWNGF	193	ND	0.00	chloroplast membrane protein ALBINO3 or ARTEMIS
92	CMT172C	MLGFTTSLQGISLRIRAPALAKCSNSKR SGAAVPAAFRLRADESSDYEQPTERR LSDALKERDVEARLAREEAERRDRAR REERLRRQRKIEELMSIPDDKEAGTIDE FL	110	ND	0.24	presumed transcriptional regulator for RuBisCO operon
93	CMT220C	MVTPGFISSTVGARGMSSRSCKALALS RIPVLVTWRRTRLNAAKHRGGLRLSQ KADDQASPSESKQKSEQSGALDGKQ QTTGVNDSEQSL	90	54	0.61	chlorophyll a synthase
94	CMT289C	MHKPAFLHIGSLSLNSGTSVRWLRPCA KTHASCCRVLVTQNPAFRVARTGTSS SHHCRLRTTKSRLFMASGQV	73	83	0.51	PGR5
95	CMT423C	MDTSGFRLVAFVPITVRGAGTLTRRCF RVQGQSPVSFDRSRTYGKCTRLAFAR ARSNNGYVMALGGDAGPKKREQQPA SEAQPSEPPSNHDGAKGADAAASSQA ENETHLTHEETTGEQEGEEEAMEGED LLNSPPFLKRKIEILEKEL	149	ND	0.14	chloroplast molecular chaperone GrpE
96	CMT579C	MATRRFSYRRVSIWNWALVAVLVHVC CCLFGRAVLVGAKDASSGGGKIE	49	38	0.00	luminal binding protein BiP
97	CMT633C	MCAFIVTGSQCSRAQANVWTKETVAC RRGPKPRSSTAKGRPLRMGVERVPPP KPGQ	56	44	0.69	ribulose-5-phosphate 3-epimerase

ND: Not detected.

Journal of Cell Science • Supplementary information

Table S4. List of putative α-helical sequences in mitochondrial TPs.

#	Gene ID	Estimated mTP length	Length of putative α-helix in mTP	Hydrophobicity (H)	Hydrophobic moment (μH)	Estimated charge	Sequence of putative α-helix	Annotation
1	CMB077C	37	25	0.553	0.4	2.9	LKGVAFFLLKGVAEL SKNATSLLKL	similar to mitochondrial inner membrane protease IMP1
2	CMC099C	25	6	ND	ND	ND	EAFCIA	NADH dehydrogenase I (Complex I) beta subcomplex 7 (B18)
3	CMC148C	33	25	0.305	0.29	3.9	VGLLTRRNLRPLQSL ARKQSQLDTF	aspartate aminotransferase
4	CMD058C	90	23	0.424	0.267	1.9	SAQRARAVSSLWAS LRPLEAYLA	fumarate hydratase precursor
5	CME198C	27	25	0.432	0.311	2.9	VRSNLGLLASCGAAY WRRLATSAGS	mitochondrial F1FO ATP synthase subunit ATP5
6	CMF062C	59	12	0.806	0.373	0.9	PYTSSSWWWRAI	mitochondrial general import pore subunit Tom40
7	CMG004C	27	25	0.37	0.346	3.9	VRSNLGLLARYGAAY WRRLATSAGS	mitochondrial F1FO ATP synthase subunit ATP5
8	CMH075C	13	8	ND	ND	ND	SADSREHK	similar to mitochondrial carrier protein
9	CMH173C	41	6	ND	ND	ND	QSTEAV	similar to mitochondrial uncoupling protein
10	CMH275C	51	25	0.51	0.198	3.1	AASLLHTWKRYLCSS CLNQALAASR	mitochondrial ribosomal protein L46
11	CMJ185C	29	24	0.41	0.418	6.1	VRGSLQRVVHKRTR WLVGVFTTLR	NADH dehydrogenase I (Complex I) flavoprotein 51kDa subunit
12	CMK031C	15	8	ND	ND	ND	GRLADLVL	cytochrome c reductase (Complex III) subunit 7(VI)
13	CMM034 C	31	18	0.46	0.306	4.4	LARTVLRTALSKVLH LHR	NADH dehydrogenase I (Complex I) iron-sulfur protein 75kDa subunit
14	CMM267 C	13	8	ND	ND	ND	SCGAASVR	NADH dehydrogenase I (Complex I) alpha subcomplex 9, mitochondrial precursor
15	CMM299 C	76	25	0.397	0.101	1.9	SNSSFCQRASVTRLV QSVQLLAPNA	dihydrolipoamide dehydrogenase
16	CMN127C	43	21	0.403	0.453	4.9	KWLRPALRTTLRLW RRQWSAE	mitochondrial F-type ATPase F1 subunit delta, precursor
17	CMN320C	83	9	0.737	0.557	0.9	VWRAVTPSL	NADH dehydrogenase I (Complex I) iron-sulfur protein 13kDa subunit
18	CMO244C	49	19	0.228	0.468	5.9	YGTLRQTVRRFRGN FLRRC	inner mitochondrial membrane protein Sco1p
19	CMO303C	42	9	0.203	0.37	0.9	REVQRAVTL	similar to mitochondrial carrier protein
20	CMP013C	26	19	0.217	0.101	-1.1	LSSLQVVGTKAAEES RLES	mitochondrial DNA replication protein YHM2
21	CMP152C	18	12	0.517	0.045	0.9	QVLTRAAFAAGV	cytochrome c reductase (Complex III) cytochrome c1 subunit
22	CMQ200C	38	25	0.606	0.288	4.1	VQLWRALLTHRGMV GRFWTLTRSTV	NADH dehydrogenase I (Complex I) iron-sulfur protein 30kDa subunit (NADH-coenzyme Q reductase)
23	CMQ258C	63	25	0.536	0.014	0.2	AGVATADAAMPALVL TRSTHSGFVL	similar to mitochondrial carrier protein
24	CMQ270C	34	25	0.499	0.193	1.8	SIACSGTVQSLRRLV SVGTLGCAQA	mitochondrial chaperonin hsp60, precursor
25	CMR073C	48	22	0.358	0.207	2.8	CLASCCRRYSITVV MNETRTN	probable mitochondrial carrier proteins; Pet8p
26	CMR289C	70	25	0.329	0.313	3.9	SLTKLSRQLWKLERT SVRSFFVERA	NADH dehydrogenase I (Complex I) iron-sulfur protein 75kDa subunit
27	CMS091C	15	5	ND	ND	ND	KDLLA	probable mitochondrial carrier protein
28	CMS372C	43	25	0.511	0.257	2.9	TLVTVARSLARALGF ARQGSIGGWY	mitochondrial acyl carrier protein precursor, NADH-ubiquinone oxidoreductase (Complex I) 9.6 kD subunit
29	CMT209C	34	25	0.268	0.259	3.1	LRGPLVAELASQVSK LGSRNQHTCK	succinate--CoA ligase (ADP- or GDP-forming), mitochondrial precursor
30	CMT234C	22	7	ND	ND	ND	RSQLLLL	cysteine desulfurase IscS, mitochondrial precursor
31	CMT434C	59	25	0.35	0.37	4.2	ALRPFARALVRSSDL LVRGRAAVHG	mitochondrial F-type ATPase F1 subunit alpha, precursor

ND: Not detected.

Journal of Cell Science • Supplementary information

Table S5. Amino acid sequence and relative scores of each synthetic TP.

Name	Position																								TargetP2.0 prediction results		Experimental result (this study)
	1	2	3	4	5	6	7	8	9	10	11	12	13	14	15	16	17	18	19	20	21	22	23	24	mTP Score	SP Score	
3-R	M	S	A	L	L	L	A	L	L	L	S	L	R	A	S	L	L	R	R	T	A	T	A	A	0.726	0.246	Mitochondrion
2-R	M	S	A	L	L	L	A	L	L	L	S	L	R	A	S	L	L	V	R	T	A	T	A	A	0.342	0.649	Mitochondrion
1-R (R2)	M	R	A	L	L	L	A	L	L	L	S	L	A	A	S	L	L	V	E	T	A	T	A	A	0.000	1.000	cytosol
1-R (R3)	M	S	R	L	L	L	A	L	L	L	S	L	A	A	S	L	L	V	E	T	A	T	A	A	0.000	1.000	NT
1-R (R4)	M	S	A	R	L	L	A	L	L	L	S	L	A	A	S	L	L	V	E	T	A	T	A	A	0.000	1.000	NT
1-R (R5)	M	S	A	L	R	L	A	L	L	L	S	L	A	A	S	L	L	V	E	T	A	T	A	A	0.000	1.000	NT
1-R (R6)	M	S	A	L	L	R	A	L	L	L	S	L	A	A	S	L	L	V	E	T	A	T	A	A	0.000	1.000	Mitochondrion
1-R (R7)	M	S	A	L	L	L	R	L	L	L	S	L	A	A	S	L	L	V	E	T	A	T	A	A	0.001	0.999	NT
1-R (R8)	M	S	A	L	L	L	A	R	L	L	S	L	A	A	S	L	L	V	E	T	A	T	A	A	0.009	0.990	Mitochondrion
1-R (R9)	M	S	A	L	L	L	A	L	R	L	S	L	A	A	S	L	L	V	E	T	A	T	A	A	0.045	0.947	Mitochondrion
1-R (R10)	M	S	A	L	L	L	A	L	L	R	S	L	A	A	S	L	L	V	E	T	A	T	A	A	0.151	0.825	NT
1-R (R11)	M	S	A	L	L	L	A	L	L	L	R	L	A	A	S	L	L	V	E	T	A	T	A	A	0.004	0.993	NT
1-R (R12)	M	S	A	L	L	L	A	L	L	L	S	R	A	A	S	L	L	V	E	T	A	T	A	A	0.090	0.869	Mitochondrion
1-R (R13)	M	S	A	L	L	L	A	L	L	L	S	L	R	A	S	L	L	V	E	T	A	T	A	A	0.019	0.971	Mitochondrion
1-R (R14)	M	S	A	L	L	L	A	L	L	L	S	L	A	R	S	L	L	V	E	T	A	T	A	A	0.010	0.985	NT
1-R (R15)	M	S	A	L	L	L	A	L	L	L	S	L	A	A	R	L	L	V	E	T	A	T	A	A	0.003	0.996	Mitochondrion
1-R (R16)	M	S	A	L	L	L	A	L	L	L	S	L	A	A	S	R	L	V	E	T	A	T	A	A	0.003	0.996	NT
1-R (R17)	M	S	A	L	L	L	A	L	L	L	S	L	A	A	S	L	R	V	E	T	A	T	A	A	0.001	0.999	NT
1-R (R18)	M	S	A	L	L	L	A	L	L	L	S	L	A	A	S	L	L	R	E	T	A	T	A	A	0.001	0.998	NT
1-R (R19)	M	S	A	L	L	L	A	L	L	L	S	L	A	A	S	L	L	V	R	T	A	T	A	A	0.000	1.000	NT
1-R (R20)	M	S	A	L	L	L	A	L	L	L	S	L	A	A	S	L	L	V	E	R	A	T	A	A	0.000	1.000	NT
1-R (R21)	M	S	A	L	L	L	A	L	L	L	S	L	A	A	S	L	L	V	E	T	R	T	A	A	0.000	1.000	Mitochondrion
1-R (R22)	M	S	A	L	L	L	A	L	L	L	S	L	A	A	S	L	L	V	E	T	A	R	A	A	0.000	1.000	NT
1-R (R23)	M	S	A	L	L	L	A	L	L	L	S	L	A	A	S	L	L	V	E	T	A	T	R	A	0.000	1.000	NT
1-R (R24)	M	S	A	L	L	L	A	L	L	L	S	L	A	A	S	L	L	V	E	T	A	T	A	R	0.000	1.000	NT
0-R	M	S	A	L	L	L	A	L	L	L	S	L	A	A	S	L	L	V	E	T	A	T	A	A	0.000	1.000	cytosol
3-K	M	S	A	L	L	L	A	L	L	L	S	L	K	A	S	L	L	K	K	T	A	T	A	A	0.099	0.848	Mitochondrion

NT: Not tested.

Journal of Cell Science • Supplementary information

Table S6. Top 50 of the peptide sequences that are similar to the synthetic TP evaluated by BLOSUM30.

#	Gene ID	Annotation	Sequence of the N-terminal polypeptide (1-24 aa)	Score
**---**	**synTP (3R)**	**Synthetic mitochondrial presequence**	**MSALLLALLLSLRASLLRRTATAA**	**103**
1	CMD051C	hypothetical protein	MFVETFAVLAGARLATLWRLRANT	41
**2**	**CMN049C**	**Formin-like protein**	**METNALALVTQRRLCIAEAVARAA**	**40**
**3**	**CMM019C**	**chloroplast ribosomal protein S1 precursor**	**MWAFLASLPLRSRSDFVSRSVRLG**	**38**
4	CMP084C	mitochondrial phosphate translocator MIR1	MSIGSASSFLVQRISLTMRQQLAS	38
5	CMK154C	hypothetical protein, conserved	MKTLWYGVLLSVFLCFFARTLQTM	37
6	CMS319C	cytochrome P450, family 51	MGTILAALANQFQALLARARDGDT	37
7	CME198C	mitochondrial F1FO ATP synthase subunit ATP5	MVRSNLGLLASCGAAYWRRLATSA	36
8	CMS047C	mitochondrial ribosomal protein L21 precursor	MRVLERVIHVLRRASPLQRASIAS	36
9	CMA007C	serine acetyltransferase (SAT)	MFATTGRLYQTLRVHPSHRALQSV	35
10	CMK256C	hypothetical protein, conserved	MSAYLGSDVLSVSVSEDGSSGRAL	35
11	CMQ239C	hypothetical protein	MSSDALANEAPVNYDLLRQLSHSL	35
12	CMG004C	mitochondrial F1FO ATP synthase subunit ATP5	MVRSNLGLLARYGAAYWRRLATSA	34
13	CMK200C	hypothetical protein, conserved	MASKIVAQLIIYGGQFLLRGLAEA	34
14	CMP334C	DnaJ (Hsp40) homolog, subfamily D	MATPLLAGIAVAGAALAGRAAIRA	34
15	CMT485C	mitochondrial ribosomal protein S7, precursor	MSLYTADSVLSKLVNILMRDGKKS	34
16	CMJ065C	CENP-C	MSAQALSLAELLRLRSLSSSGTSL	33
17	CMO202C	coatomer protein complex, subunit delta	MVVLAIAILNKLGRLLISRQFREM	33
18	CMR062C	hypothetical protein, conserved	MGTDTNSEDSAKRFRRVRRGSATF	33
19	CMT290C	similar to TFIIH subunit SSL1	MTQLCVERSGSCRLHLQRPVQQSA	32
20	CMF124C	hypothetical protein, conserved	MLSESLQSFLKLRTAPLVLVAATP	31
21	CMF141C	similar to prefoldin subunit3	MKLKLFNATAHLRPAICRQCRRDV	31
22	CMK272C	hypothetical protein	MSTDVLVTADTTLADLAWPIVSAA	31
23	CML069C	hypothetical protein	MEELLREQVFNAIVSFLQRERLPA	31
24	CML165C	hypothetical protein	MGSRTPATGTALFDSVLRLAREKS	31
25	CMQ203C	hypothetical protein	MWASVLRFLPKHKGNLGRCMSVRA	31
26	CMS090C	hypothetical protein, conserved	MTLFFVTCVGSASISGLQRSSTAR	31
27	CMC145C	60S ribosomal protein L9	MRAILASETLKVPEGVTVRVKKRE	30
28	CME158C	similar to UGA suppressor tRNA-associated antigenic protein	MDADLLEKARALADPAYVTVAEAA	30
29	CMN338C	NADPH:protochlorophyllide oxidoreductase, chloroplast precursor	MQALFVSVPLYQERYIVRARAATR	30
30	CMP030C	probable chromatin assembly factor 1 subunit B	MRALVLELEWHEQQRGVFSVDIAA	30
31	CMS296C	probable C-4 sterol methyl oxidase	MILEFTVIAIATRWSAIQRLWLAV	30
32	CMT440C	hypothetical protein	MATGTTGARVLSMASILRHRASLP	30
33	CMC133C	similar to oxygen-evolving complex component psbQ	MFVTSFQLQPKGKGSFLARSAGSR	29
34	CMI142C	dihydroxy-acid dehydratase	MSNHTLQFLTTTETSFVKGSFPRD	29
35	CML189C	hypothetical protein	MWSLPVPLASNSRLAERRKLASHA	29
36	CMM267C	NADH dehydrogenase I (Complex I) alpha subcomplex 9, mitochondrial precursor	MDSCGAASVRLLRLSLQRNGSAVR	29
37	CMN294C	hypothetical protein	MASLAAVVRALLRRESQRATGYAL	29
38	CMP057C	hypothetical protein, conserved	MRADTVAFLVWSGLGPNSRQPRAS	29
39	CMQ211C	adenylosuccinate lyase	MLTLAFAFSAAPSFTSLRKSSHRG	29
40	CMS141C	hypothetical protein	MSTDLEELSAAWQHRLLEEFTVVD	29
41	CMS212C	mitochondrial ribosomal protein S17 precursor	MYSALTALTRIVRRNVLSLASASG	29
42	CMT598C	similar to heterogeneous nuclear ribonucleoprotein	MQALVGALRPTLAGPLDQHTDIRD	29
43	CMB053C	probable xylulose kinase	MVAESNGLYLGLDLSTQSLDAVVI	28
44	CMF014C	hypothetical protein	MAANTEGDEVSLPVAKTLRSILEL	28
45	CMF129C	mitochondrial chaperonin hsp10, precursor	MSALSRRILPLLDRVLVEKIVPKK	28
46	CMH102C	similar to splicing factor 3a, subunit 2	MDAYALKNRLGAVECKLCRTVHPS	28
47	CMJ066C	phenylalanine hydroxylase	MSSTVLRLLSPYSNIVERSSGLFR	28
48	CMK199C	mutS family DNA mismatch repair protein MSH4	MSRLVTGSSRSLRTLSKRSGTFAS	28
49	CMK309C	unknown zinc-finger protein	MLLLVVEVVYGKGRSLLQQAQCMH	28
50	CMN095C	similar to splicing factor 3a subunit 2, partial	MDAYALKNRLGAVECKLCRTVHPS	28

Journal of Cell Science • Supplementary information

Table S7. Primers and synthetic DNA fragments used in this study.

#	Forward or reverse	Sequence	Product
1	F	TAAACTAGCTATTTATCTGGTACATATCATTCATAAGCACATG	Vector
2	R	ATCAGAAGTGCTTTACGAGGAACGTTCAG	
3	F1	ACCCTTGCAGTCACTCGCCCGGAAACAAAGCCAGTTGGACACGTTTCGGAGCCGCATGTCTTATGTTAGCAAGGGCGAAGAGCTGTTTAC	AAT (1-33 aa) and mVenus
4	F2	CCTCGTAAAGCACTTCTGATATGTTTGTGGGACTTCTCACAAGACGCAATCTACGACCCTTGCAGTCACTCGCCC	
5	R	ATGTACCAGATAAATAGCTAGTTTACTTATACAGTTCATCCATACCCAGCGTAATGC	
6	F1	TGTCGCTGCGCGCATCGCTGCTCCGCCGCACGGCGACGGCGGCCGTTAGCAAGGGCGAAGAGCTGTTTAC	synTP^{3R} and mVenus
7	F2	CCTCGTAAAGCACTTCTGATATGAGCGCACTGCTCCTGGCACTGCTCCTGTCGCTGCGCGCATCGCTGCTCCG	
8	R	ATGTACCAGATAAATAGCTAGTTTACTTATACAGTTCATCCATACCCAGCGTAATGC	
9	F1	GTCGCTGCGCGCATCGCTGCTCGTGCGCACGGCGACGGCGGCCGTTAGCAAGGGCGAAGAGCTGTTTAC	synTP^{2R} and mVenus
10	F2	CCTCGTAAAGCACTTCTGATATGAGCGCACTGCTCCTGGCACTGCTCCTGTCGCTGCGCGCATCG	
11	R	ATGTACCAGATAAATAGCTAGTTTACTTATACAGTTCATCCATACCCAGCGTAATGC	
12	F1	GTCGCTGCGCGCATCGCTGCTCGTGGAGACGGCGACGGCGGCCGTTAGCAAGGGCGAAGAGCTGTTTAC	synTP^{1R} (R13) and mVenus
13	F2	CCTCGTAAAGCACTTCTGATATGAGCGCACTGCTCCTGGCACTGCTCCTGTCGCTGCGCGCATCG	
14	R	ATGTACCAGATAAATAGCTAGTTTACTTATACAGTTCATCCATACCCAGCGTAATGC	
15	F1	TGTCGCTGGCGGCATCGCTGCTCGTGGAGACGGCGACGGCGGCCGTTAGCAAGGGCGAAGAGCTGTTTAC	synTP^{0R} and mVenus
16	F2	CCTCGTAAAGCACTTCTGATATGAGCGCACTGCTCCTGGCACTGCTCCTGTCGCTGGCGGCATCG	
17	R	ATGTACCAGATAAATAGCTAGTTTACTTATACAGTTCATCCATACCCAGCGTAATGC	
18	F1	GCTGAAGGCATCGCTGCTCAAGAAAACGGCGACGGCGGCCGTTAGCAAGGGCGAAGAGCTGTTTAC	synTP^{3K} and mVenus
19	F2	CCTCGTAAAGCACTTCTGATATGAGCGCACTGCTCCTGGCACTGCTCCTGTCGCTGAAGGCATCGCTGCTCAAG	
20	R	ATGTACCAGATAAATAGCTAGTTTACTTATACAGTTCATCCATACCCAGCGTAATGC	
21	F1	GGTTACGCAGCGGAGGCTCTGTATTGCTGAGGCAGTTGCTCGGGCTGCGGTTAGCAAGGGCGAAGAGCTGTTTAC	FMNL (1-24 aa) and mVenus
22	F2	CCTCGTAAAGCACTTCTGATATGGAAACGAATGCTTTGGCTCTGGTTACGCAGCGGAGGCTCTGTATTG	
23	R	ATGTACCAGATAAATAGCTAGTTTACTTATACAGTTCATCCATACCCAGCGTAATGC	
24	F1	GGTTACGCAGCGGAGGCTCTGTATTGCTGAGGCAGTTGCTCGGGCTGCGGTTAGCAAGGGCGAAGAGCTGTTTAC	FMNL 1-24 (E2A) and mVenus
25	F2	CCTCGTAAAGCACTTCTGATATGGCCACGAATGCTTTGGCTCTGGTTACGCAGCGGAGGCTCTGTATTG	
26	R	ATGTACCAGATAAATAGCTAGTTTACTTATACAGTTCATCCATACCCAGCGTAATGC	
27	F1	GGTTACGCAGCGGAGGCTCTGTATTGCTGCCGCAGTTGCTCGGGCTGCGGTTAGCAAGGGCGAAGAGCTGTTTAC	FMNL 1-24 (E18A) and mVenus
28	F2	CCTCGTAAAGCACTTCTGATATGGAAACGAATGCTTTGGCTCTGGTTACGCAGCGGAGGCTCTGTATTG	
29	R	ATGTACCAGATAAATAGCTAGTTTACTTATACAGTTCATCCATACCCAGCGTAATGC	
30	F1	GGTTACGCAGCGGAGGCTCTGTATTGCTGCCGCAGTTGCTCGGGCTGCGGTTAGCAAGGGCGAAGAGCTGTTTAC	FMNL 1-24 (E2A/E18A) and mVenus
31	F2	CCTCGTAAAGCACTTCTGATATGGCCACGAATGCTTTGGCTCTGGTTACGCAGCGGAGGCTCTGTATTG	
32	R	ATGTACCAGATAAATAGCTAGTTTACTTATACAGTTCATCCATACCCAGCGTAATGC	
33	F1	GCGCAGTCGCAGTGACTTTGTGTCCCGCTCGGTGCGGTTGGGGGTTAGCAAGGGCGAAGAGCTGTTTAC	RPSA (1-24 aa) and mVenus
34	F2	CCTCGTAAAGCACTTCTGATATGTGGGCGTTTCTTGCGAGTCTTCCATTGCGCAGTCGCAGTGACTTTGTG	
35	R	ATGTACCAGATAAATAGCTAGTTTACTTATACAGTTCATCCATACCCAGCGTAATGC	
36	F	GTTAGCAAGGGCGAAGAGCTGTTTAC	Vector for AAT or RPSA full-length
37	R	ATCAGAAGTGCTTTACGAGGAACGTTCAG	
38	F	CCTCGTAAAGCACTTCTGATATGTTTGTGGGACTTCTCACAAGACGCAATCTACGACCCTTGCAGTCACTCGCCC	AAT full-length
39	R	GTAAACAGCTCTTCGCCCTTGCTAACTATCTTACCGCTGTTTAGCGCAGCTTG	
40	F	CCTCGTAAAGCACTTCTGATATGTGGGCGTTTCTTGCGAGTCTTCCATTGCGCAGTCGCAGTGACTTTGTG	RPSA full-length
41	R	TCTTCGCCCTTGCTAACGGAAGATGCCGGAGAGGATTCTTCC	
42	F	TACGGCAAGCTGACGCTCAAGC	Vector for cloning of synthetic DNA fragment
43	R	GAATCTCGACCCACGTCCGTCG	
44	---	ACGTGGGTCGAGATTCGATTTCGGAGGCCGCACGACGCAAAAAGGTCATTCGAGTTTGTGTCTGCGGACTCTGAACGTTCCTCGTAAAGCACTTCTGATATGCGCGCGCTGCTCCTAGCTCTCCTTCTCAGCTTGGCGGCATCGCTGCTGGTGGAGACGGCCACCGCAGCCGTTAGCAAGGGCGAAGAGCTGTTTACGGGCGTGGTCCCAATCCTGGTGGAACTCGACGGCGATGTCAACGGTCACAAATTCTCGGTGTCGGGCGAGGGCGAGGGTGATGCAACCTACGGCAAGCTGACG	Synthetic DNA of synTP^{1R} (R2)
45	---	ACGTGGGTCGAGATTCGATTTCGGAGGCCGCACGACGCAAAAAGGTCATTCGAGTTTGTGTCTGCGGACTCTGAACGTTCCTCGTAAAGCACTTCTGATATGAGCGCACTGCTACGCGCGCTGCTTCTGTCCTTGGCGGCCTCGCTCCTCGTGGAGACCGCAACGGCCGCTGTTAGCAAGGGCGAAGAGCTGTTTACGGGCGTGGTCCCAATCCTGGTGGAACTCGACGGCGATGTCAACGGTCACAAATTCTCGGTGTCGGGCGAGGGCGAGGGTGATGCAACCTACGGCAAGCTGACG	Synthetic DNA of synTP^{1R} (R6)

Journal of Cell Science • Supplementary information

46	---	ACGTGGGTCGAGATTCGATTTCGGAGGCCGCACGACGCAAAAAGGTCATTCGAGTTTGTGTCTGCGGAC<br>TCTGAACGTTCCTCGTAAAGCACTTCTGATATGTCCGCACTCCTGTTGGCTCGCCTGCTCTCGCTTGCAG<br>CGAGCCTACTGGTGGAGACGGCGACCGCCGCCGTTAGCAAGGGCGAAGAGCTGTTTACGGGCGTGGTC<br>CCAATCCTGGTGGAACTCGACGGCGATGTCAACGGTCACAAATTCTCGGTGTCGGGCGAGGGCGAGGG<br>TGATGCAACCTACGGCAAGCTGACG	Synthetic DNA of synTP^{1R} (R8)
47	---	ACGTGGGTCGAGATTCGATTTCGGAGGCCGCACGACGCAAAAAGGTCATTCGAGTTTGTGTCTGCGGAC<br>TCTGAACGTTCCTCGTAAAGCACTTCTGATATGAGCGCCCTATTGCTCGCGCTGCGCCTTTCGCTGGCCG<br>CGTCCCTGCTCGTGGAGACCGCAACGGCAGCTGTTAGCAAGGGCGAAGAGCTGTTTACGGGCGTGGTC<br>CCAATCCTGGTGGAACTCGACGGCGATGTCAACGGTCACAAATTCTCGGTGTCGGGCGAGGGCGAGGG<br>TGATGCAACCTACGGCAAGCTGACG	Synthetic DNA of synTP^{1R} (R9)
48	---	ACGTGGGTCGAGATTCGATTTCGGAGGCCGCACGACGCAAAAAGGTCATTCGAGTTTGTGTCTGCGGAC<br>TCTGAACGTTCCTCGTAAAGCACTTCTGATATGAGCGCTCTTCTATTGGCCCTGCTCCTCTCGCGCGCAG<br>CATCCCTGCTGGTGGAGACCGCGACGGCCGCGGTTAGCAAGGGCGAAGAGCTGTTTACGGGCGTGGTC<br>CCAATCCTGGTGGAACTCGACGGCGATGTCAACGGTCACAAATTCTCGGTGTCGGGCGAGGGCGAGGG<br>TGATGCAACCTACGGCAAGCTGACG	Synthetic DNA of synTP^{1R} (R12)
49	---	ACGTGGGTCGAGATTCGATTTCGGAGGCCGCACGACGCAAAAAGGTCATTCGAGTTTGTGTCTGCGGAC<br>TCTGAACGTTCCTCGTAAAGCACTTCTGATATGTCGGCTCTCCTACTCGCACTGCTTTTGAGCCTGGCGG<br>CCCGCCTGCTCGTGGAGACGGCAACCGCCGCGGTTAGCAAGGGCGAAGAGCTGTTTACGGGCGTGGTC<br>CCAATCCTGGTGGAACTCGACGGCGATGTCAACGGTCACAAATTCTCGGTGTCGGGCGAGGGCGAGGG<br>TGATGCAACCTACGGCAAGCTGACG	Synthetic DNA of synTP^{1R} (R15)
50	---	ACGTGGGTCGAGATTCGATTTCGGAGGCCGCACGACGCAAAAAGGTCATTCGAGTTTGTGTCTGCGGAC<br>TCTGAACGTTCCTCGTAAAGCACTTCTGATATGTCGGCGCTGCTCCTAGCACTTCTGCTCAGCCTGGCCG<br>CTTCCTTGCTCGTGGAGACGCGCACCGCGGCCGTTAGCAAGGGCGAAGAGCTGTTTACGGGCGTGGTC<br>CCAATCCTGGTGGAACTCGACGGCGATGTCAACGGTCACAAATTCTCGGTGTCGGGCGAGGGCGAGGG<br>TGATGCAACCTACGGCAAGCTGACG	Synthetic DNA of synTP^{1R} (R21)
51	F	CTTCAAGAAAAGAGGATCTTTTGCCGTG	For transformation
52	R	CCCTAGCAGCTGACTGTATCTCTATTC	

Journal of Cell Science • Supplementary information
