# Supplementary material for: Simple prerequisite of presequence for mitochondrial protein import in the unicellular red alga Cyanidioschyzon merolae
Source: J Cell Sci. 2024 Jul 23;137(14):jcs262042. doi: 10.1242/jcs.262042 (PMC11298712; doi:10.1242/jcs.262042)
Supplement: Supplementary file 1 [file joces-137-262042_review_history.pdf]

Journal of Cell Science

Simple prerequisite of presequence for mitochondrial protein import in the unicellular red alga *Cyanidioschyzon merolae*

Riko Hirata, Yuko Mogi, Kohei Takahashi, Hisayoshi Nozaki, Tetsuya Higashiyama and Yamato Yoshida
DOI: 10.1242/jcs.262042

Editor: Jennifer Lippincott-Schwartz

Review timeline
Original submission: 19 February 2024
Accepted: 13 June 2024

Original submission

First decision letter

MS ID#: JOCES/2024/262042

MS TITLE: Simple prerequisite of presequence for mitochondrial protein import in the unicellular red alga *Cyanidioschyzon merolae*

AUTHORS: Riko Hirata, Yuko Mogi, Kohei Takahashi, Hisayoshi Nozaki, Tetsuya Higashiyama, and Yamato Yoshida
ARTICLE TYPE: Research Article

I am happy to tell you that your manuscript has been accepted for publication in Journal of Cell Science, pending standard ethics checks.

Reviewer 1

Advance summary and potential significance to field

In Harata et al, the authors use a combination of bioinformatics, microscopy, and cell biology to study mitochondrial targeting peptides in Cyanidioschyzon merolae, an alga containing a single mitochondrion. With fusions to mVenus, the authors deduce a simple formula for a synthetic mitochondrial targeting peptide in this species. Using a series of expression constructs and side-directed mutagenesis, the authors present a framework where a short sequence (24 amino acids) that is mostly hydrophobic, has at least one basic amino acid (not in position 2), minimal to no negative charges, and is often alpha helical is typically permeable to the C. merolae mitochondrion. Addition of additional negative charges (aspartate or glutamate) or absence of any positive charges (arginine or lysine tested) interferes with mitochondrial targeting.

As further evidence, the authors show that RPSA, a probable chloroplast ribosomal protein, has a leader sequence predictive of a mitochondrial target peptide. If the first 24 amino acids of this protein are fused with mVenus, the protein is mitochondrial. If full length protein is fused to mVenus, RPSA becomes chloroplast localized. It is speculated that additional sequences within chloroplast sequences target them away from the mitochondria, which the bioinformatic data support as chloroplast targeting sequences are typically longer than mitochondrial. Similar support is provided for a non-mitochondrial protein where neutralization of negative charges in the first 24 amino acids can transform the sequence into a mitochondrial targeting sequence.

Comments for the author

© 2024. Published by The Company of Biologists under the terms of the Creative Commons Attribution License (https://creativecommons.org/licenses/by/4.0/).

The evidence is convincing, and the study is thorough, however, in its present form the study is limited to C. merolae. It is not tested if this synthetic mitochondrial targeting peptide has any applicability to the most common model systems used in cell biology (human, mouse, drosophila, yeast). The study is concise, very well written, and I have no major concerns regarding the datasets.

Major comment:

The study is limited by its use of a single alga species and lack of verification in organisms more commonly used in cell biology. Thus, the minimalist mitochondrial targeting peptide presented here may not apply to higher level organisms. For example, Halo-MTS (Addgene #124315), a mitochondrial targeted HaloTag used in our lab for mammalian cell lines, has a 66 amino acid mitochondrial targeting sequence containing 3 lysines and 6 arginines (with no acidic residues). Would a 24 amino acid targeting sequence with a single positively charged residue target to the mammalian mitochondria? Given the substantial charge difference between Human TOM22 and C. merolae TOM22 (Fig. 6), one may hypothesize that the targeting sequences should be different, and the human one may require more positive charges. At present, the scope of the study is extremely small, and it's unclear if this study reaches the threshold of "be of broad interest to the cell biology community" requirement for publication in JCS.

Minor comments:

1) Why was the ACTIN knockout cell used for imaging? Please include in methods.

2) What were mScarlet and mCerulean3 fused to for visualization of mitochondrial and peroxisome?

3) Was histidine tested as a suitable positive charge carrier?

4) Lines 13 and 16, page 25: spelling of sequence.

<u>Reviewer 2</u>

Advance summary and potential significance to field

The paper by Yoshida and colleagues presents new results characterizing mitochondrial protein import in red alga. Using compelling fluorescent imaging and molecular biology approaches, the authors define the requirements for protein import into mitochondria in C. merolae and design a synthetic targeting peptide to show the minimal requirements for such import. Their results show that the leader sequence for import into mitochondria must have at least one basic residue. Notably, no other specificity is required such as net charge, hydrophobicity ad hydrophobic content needed for import in animal and fungi. Based on their findings, the authors conclude that compared to animal and fungi cells, which use a complex mechanism for protein import into mitochondria, C. merolae use a simple single-step authentication. The results are very compelling and nicely illustrated. Given the paucity in our understanding of mitochondria import in alga, the findings are worth publishing.

Comments for the author

The paper by Yoshida and colleagues presents new results characterizing mitochondrial protein import in red alga. Using compelling fluorescent imaging and molecular biology approaches, the authors define the requirements for protein import into mitochondria in C. merolae and design a synthetic targeting peptide to show the minimal requirements for such import. Their results show that the leader sequence for import into mitochondria must have at least one basic residue. Notably, no other specificity is required such as net charge, hydrophobicity ad hydrophobic content needed for import in animal and fungi. Based on their findings, the authors conclude that compared to animal and fungi cells, which use a complex mechanism for protein import into mitochondria, C. merolae use a simple single-step authentication. The results are very compelling and nicely

© 2024. Published by The Company of Biologists under the terms of the Creative Commons Attribution License (https://creativecommons.org/licenses/by/4.0/).

illustrated. Given the paucity in our understanding of mitochondria import in alga, the findings are worth publishing.

© 2024. Published by The Company of Biologists under the terms of the Creative Commons Attribution License (https://creativecommons.org/licenses/by/4.0/).
